# Supplementary material for: Benchmarking uncertainty quantification for protein engineering
Source: PLoS Comput Biol. 2025 Jan 7;21(1):e1012639. doi: 10.1371/journal.pcbi.1012639 (PMC11741572; doi:10.1371/journal.pcbi.1012639)
Supplement: S1 Appendix — Code availability, OHE results, OHE vs. ESM comparison, additional prediction and uncertainty evaluation metrics, and additional active learning results. (PDF) [file pcbi.1012639.s001.pdf]

Supporting Information
Benchmarking Uncertainty Quantification for Protein Engineering

Kevin P. Greenman,[†,¶] Ava P. Amini,[*,‡] and Kevin K. Yang[*,‡]

†*Department of Chemical Engineering, Massachusetts Institute of Technology, Cambridge, MA, USA*

‡*Microsoft Research New England, Cambridge, MA, USA*

¶*Work done in part during an internship at Microsoft Research*

E-mail: ava.amini@microsoft.com; yang.kevin@microsoft.com

1 Code Availability

The code for the models, uncertainty methods, and evaluation metrics in this work is available at `https://github.com/microsoft/protein-uq` and archived at `https://zenodo.org/doi/10.5281/zenodo.7839141`.

2 OHE Results

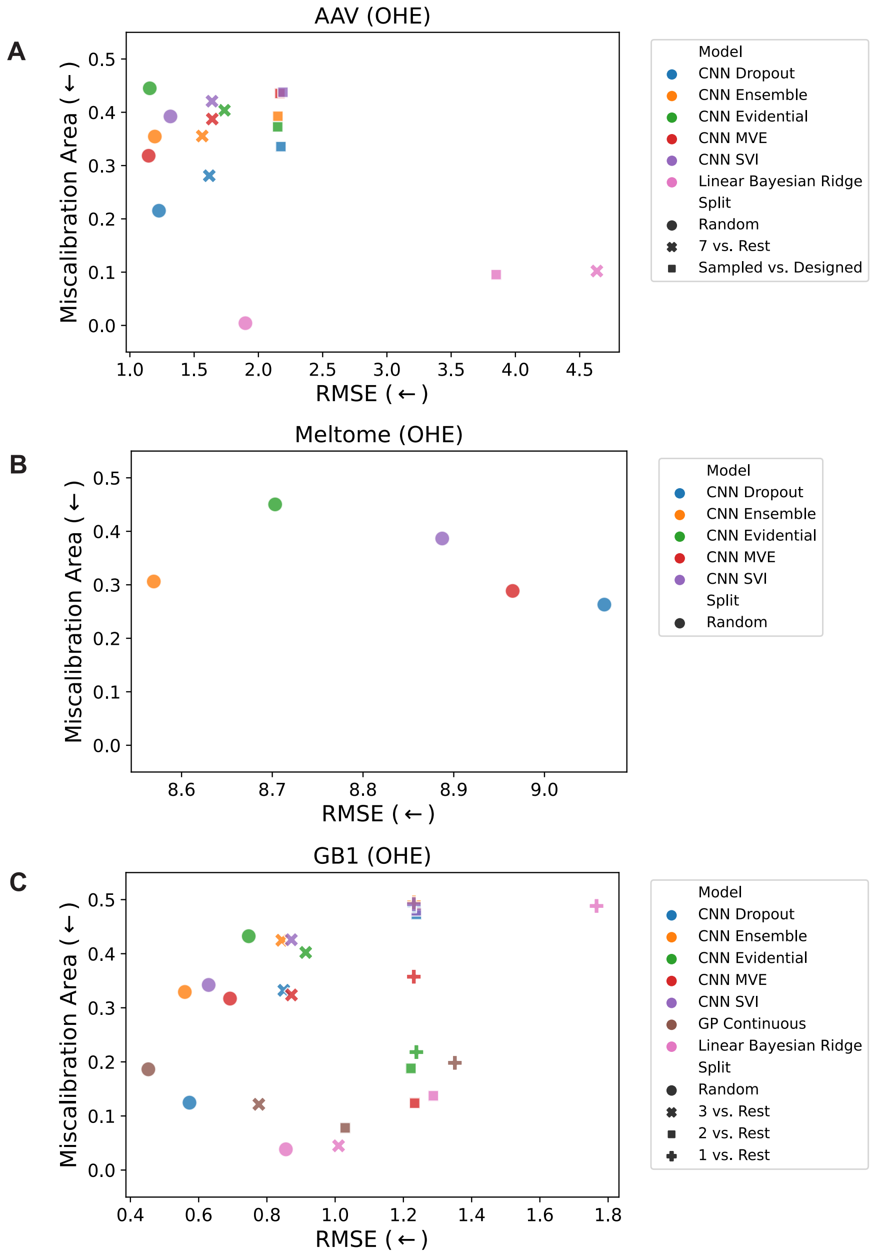

Figure A: Miscalibration area vs. root mean square error (RMSE) for the (a) AAV, (b) Meltome, and (c) GB1 landscapes. Miscalibration area (also called the area under the calibration error curve or AUCE) quantifies the absolute difference between the calibration plot and perfect calibration. It is desirable to have a model that is both accurate and well-calibrated, so the best performing points are those closest to the lower left corner of the plots. The GP Continuous model is not shown for the AAV landscape due to memory constraints for training these models. The GP Continuous and Linear Bayesian Ridge models are not shown for the Meltome landscape due to memory constraints and limitations of 32-bit LAPACK, respectively.

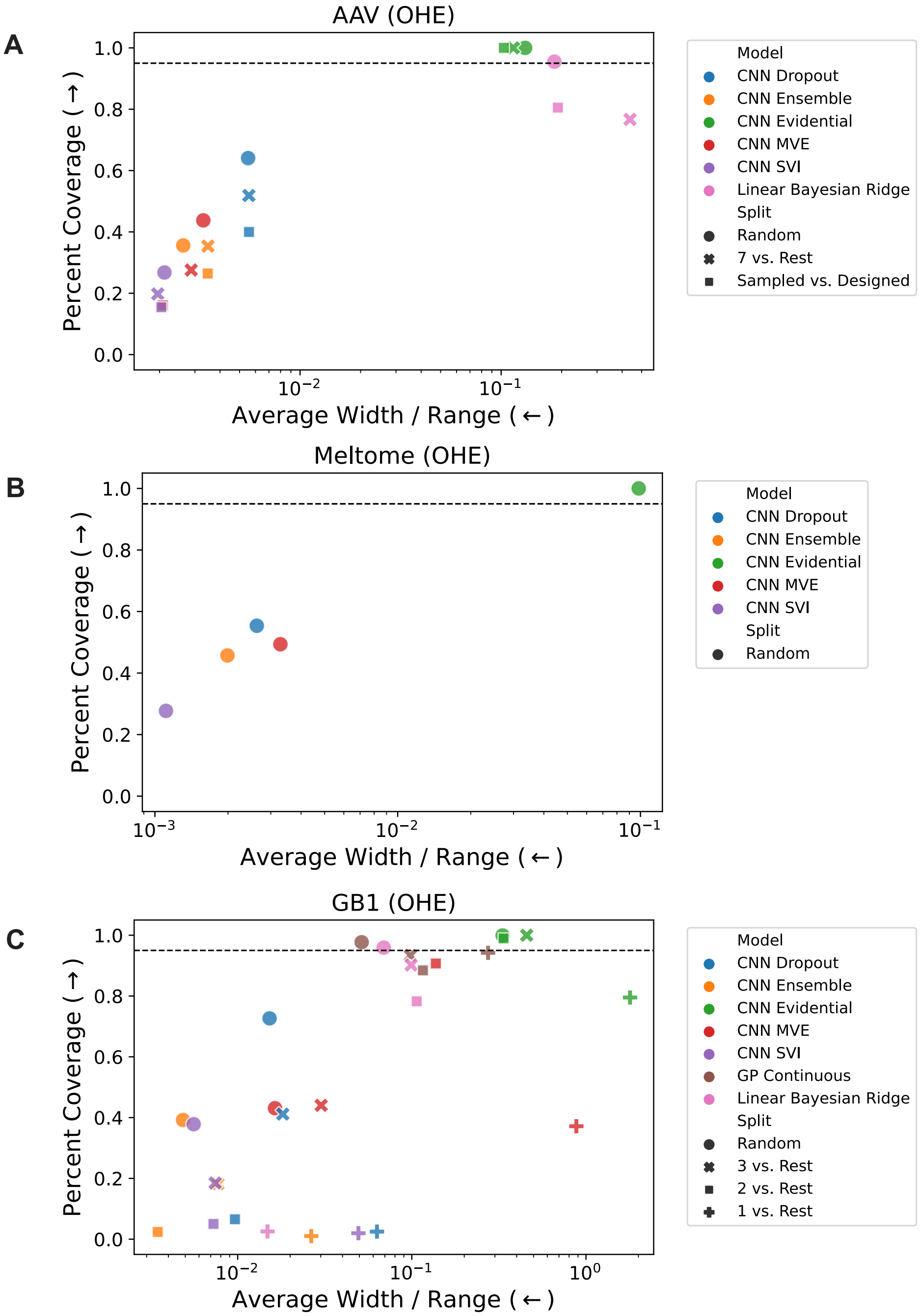

Figure B: Coverage vs. average width / range for the (a) AAV, (b) Meltome, and (c) GB1 landscapes. Coverage is the percentage of true values that fall within the 95% confidence interval ($\pm 2\sigma$) of each prediction, and the width is the size of the 95% confidence region relative to the range of the training set ($4\sigma/R$ where R is the range of the training set). A good model exhibits high coverage and low width, which corresponds to the upper left of each plot. The horizontal dashed line indicates 95% coverage. The GP Continuous model is not shown for the AAV landscape due to memory constraints for training these models. The GP Continuous and Linear Bayesian Ridge models are not shown for the Meltome landscape due to memory constraints and limitations of 32-bit LAPACK, respectively.

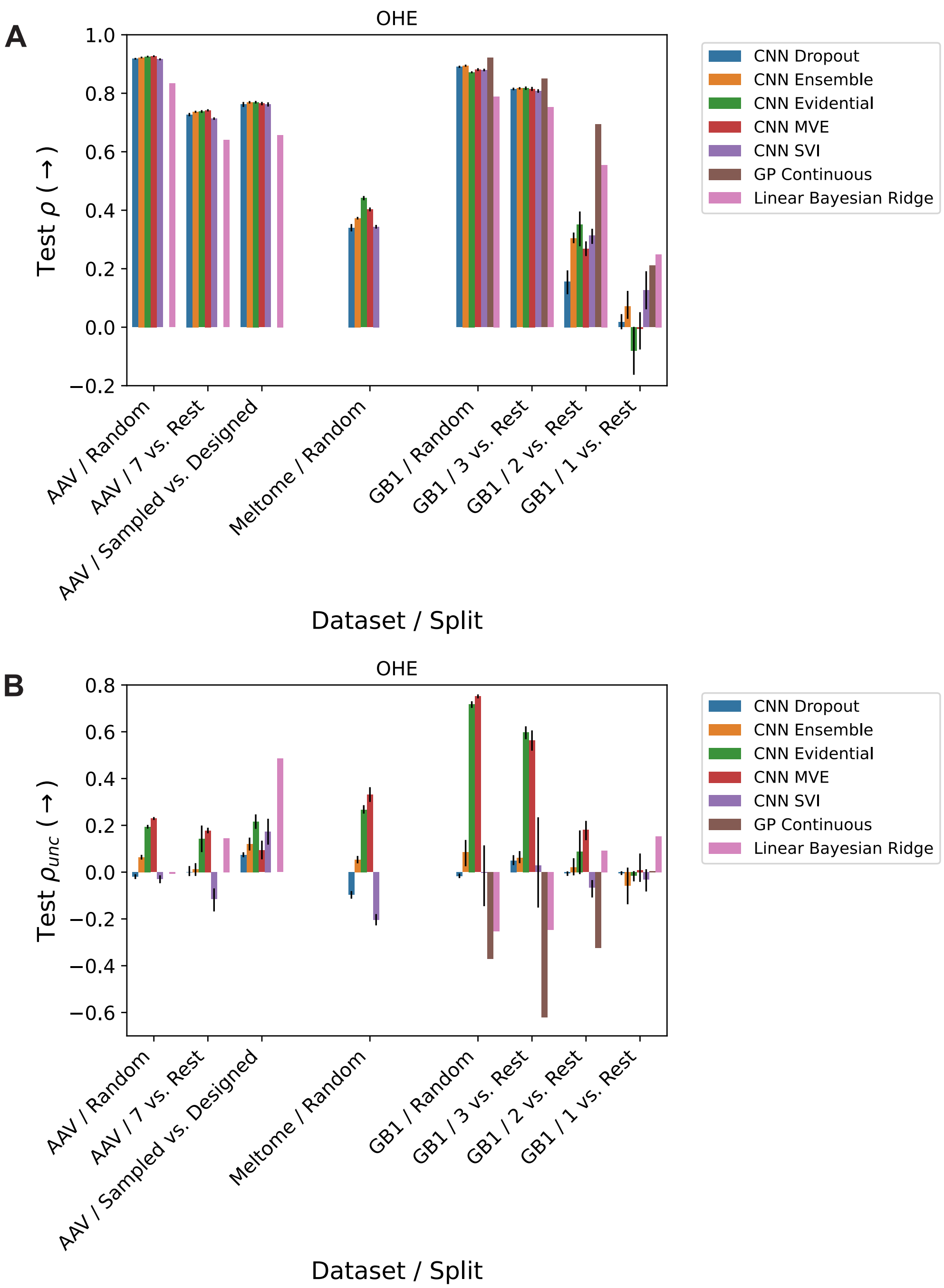

Figure C: Spearman rank correlations of (a) predictions (ρ) and (b) uncertainties (ρ_{unc}) vs. extrapolation. Within each landscape (AAV, Meltome, and GB1), splits are qualitatively ordered by the amount of domain shift between train and test sets, with the lowest domain shift on the left and the highest domain shift on the right. Error bars on the CNN results represent the 95% confidence interval calculated from 5 different random initializations of the CNN parameters.

3 OHE vs. ESM Comparison

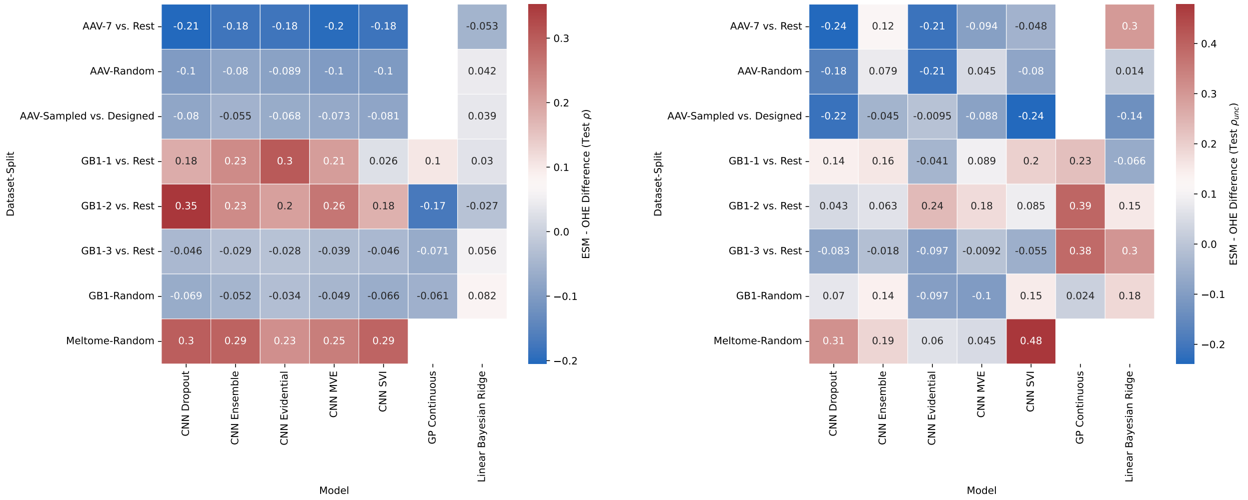

Figure D: Comparison of prediction (ρ) and uncertainty (ρ_{unc}) performance between the OHE and ESM representations across all models and tasks. Red cells indicate that the ESM representation performed better, while blue cells indicate that the OHE representation performed better.

4 Prediction and Uncertainty Evaluation Metrics

We calculate the average negative log likelihood as:

$$\overline{NLL} = -\frac{1}{2N}\sum_{i=1}^{N} \log(2\pi\sigma_i^2) + \frac{(\hat{y}_i - y_i)^2}{\sigma_i^2} \tag{1}$$

where N is the number of samples, σ_i^2 is the variance of the i-th prediction (clamped to a minimum value of 1×10^{-6}), $\hat{y}_i$ is the i-th prediction, and y_i is the i-th target. The average optimal negative log likelihood is defined as the average log likelihood if the uncertainties (standard deviations of the predictions) were exactly equal to the residual for all samples:

$$\overline{NLL_{opt}} = -\frac{1}{2N}\sum_{i=1}^{N} \log(2\pi(\hat{y}_i - y_i)^2) + 1 \tag{2}$$

Tables A-AR show the prediction and uncertainty evaluation metrics along with their

standard deviations for all landscapes, splits, and models. "Odd-numbered" tables (e.g. A, C, E, etc.) show the average metrics, while "even-numbered" tables (e.g. B, D, F, etc.) show the standard deviations from training 5 models per split with different seeds for initialization of the CNN parameters and batching / stochastic gradient descent. The standard deviations for Gaussian Process (GP) and Bayesian Ridge Regression (BRR) are always `NaN` because these models do not depend on an initial seed for reproducibility.

Table A: Test set RMSE for models trained on OHE representation (↓)

Dataset	Model<br>Split	Dropout	Ensemble	Evidential	MVE	SVI	GP	BRR
AAV	7 vs. Rest	1.618	1.563	1.736	1.641	1.639	NaN	4.634
	Random	1.226	1.194	1.155	1.146	1.316	NaN	1.899
	Sampled vs. Designed	2.175	2.152	2.150	2.168	2.191	NaN	3.850
GB1	1 vs. Rest	1.231	1.231	1.238	1.231	1.231	1.351	1.766
	2 vs. Rest	1.238	1.237	1.222	1.233	1.238	1.029	1.288
	3 vs. Rest	0.850	0.844	0.914	0.872	0.872	0.776	1.010
	Random	0.573	0.559	0.747	0.692	0.629	0.453	0.856
Meltome	Random	9.066	8.569	8.703	8.965	8.887	NaN	NaN

Table B: Test set RMSE standard deviation for models trained on OHE representation

Dataset	Model<br>Split	Dropout	Ensemble	Evidential	MVE	SVI	GP	BRR
AAV	7 vs. Rest	0.041	0.006	0.051	0.040	0.124	NaN	NaN
	Random	0.007	0.011	0.007	0.010	0.130	NaN	NaN
	Sampled vs. Designed	0.033	0.017	0.025	0.039	0.027	NaN	NaN
GB1	1 vs. Rest	0.001	0.001	0.004	0.000	0.002	NaN	NaN
	2 vs. Rest	0.002	0.001	0.022	0.001	0.004	NaN	NaN
	3 vs. Rest	0.006	0.004	0.009	0.022	0.007	NaN	NaN
	Random	0.007	0.004	0.013	0.010	0.017	NaN	NaN
Meltome	Random	0.160	0.030	0.053	0.081	0.046	NaN	NaN

Table C: Test set MAE for models trained on OHE representation (↓)

Dataset	Model<br>Split	Dropout	Ensemble	Evidential	MVE	SVI	GP	BRR
AAV	7 vs. Rest	1.274	1.229	1.350	1.268	1.311	NaN	3.364
	Random	0.937	0.909	0.863	0.863	1.017	NaN	1.499
	Sampled vs. Designed	1.720	1.682	1.670	1.703	1.732	NaN	2.676
GB1	1 vs. Rest	0.908	0.909	0.903	0.909	0.909	1.161	1.472
	2 vs. Rest	0.990	0.986	0.913	0.977	0.984	0.675	1.002
	3 vs. Rest	0.617	0.609	0.599	0.592	0.630	0.511	0.780
	Random	0.400	0.382	0.461	0.431	0.438	0.302	0.648
Meltome	Random	7.019	6.597	6.539	6.795	6.845	NaN	NaN

Table D: Test set MAE standard deviation for models trained on OHE representation

Dataset	Model<br>Split	Dropout	Ensemble	Evidential	MVE	SVI	GP	BRR
AAV	7 vs. Rest	0.038	0.003	0.034	0.028	0.114	NaN	NaN
	Random	0.007	0.009	0.007	0.009	0.122	NaN	NaN
	Sampled vs. Designed	0.029	0.006	0.017	0.014	0.021	NaN	NaN
GB1	1 vs. Rest	0.001	0.001	0.003	0.001	0.002	NaN	NaN
	2 vs. Rest	0.003	0.003	0.022	0.004	0.014	NaN	NaN
	3 vs. Rest	0.004	0.004	0.009	0.018	0.008	NaN	NaN
	Random	0.007	0.003	0.005	0.004	0.021	NaN	NaN
Meltome	Random	0.152	0.007	0.016	0.040	0.038	NaN	NaN

Table E: Test set R^2 for models trained on OHE representation ($\uparrow$)

Dataset	Model<br>Split	Dropout	Ensemble	Evidential	MVE	SVI	GP	BRR
AAV	7 vs. Rest	0.366	0.409	0.271	0.348	0.347	NaN	-4.195
	Random	0.841	0.850	0.859	0.861	0.816	NaN	0.620
	Sampled vs. Designed	0.608	0.616	0.617	0.610	0.602	NaN	-0.229
GB1	1 vs. Rest	-0.016	-0.015	-0.027	-0.015	-0.015	-0.223	-1.090
	2 vs. Rest	-0.012	-0.010	0.013	-0.004	-0.012	0.300	-0.095
	3 vs. Rest	0.556	0.562	0.487	0.532	0.533	0.629	0.373
	Random	0.774	0.785	0.616	0.670	0.727	0.859	0.496
Meltome	Random	0.391	0.456	0.439	0.405	0.415	NaN	NaN

Table F: Test set R^2 standard deviation for models trained on OHE representation

Dataset	Model<br>Split	Dropout	Ensemble	Evidential	MVE	SVI	GP	BRR
AAV	7 vs. Rest	0.032	0.005	0.043	0.032	0.103	NaN	NaN
	Random	0.002	0.003	0.002	0.002	0.038	NaN	NaN
	Sampled vs. Designed	0.012	0.006	0.009	0.014	0.010	NaN	NaN
GB1	1 vs. Rest	0.002	0.002	0.007	0.001	0.003	NaN	NaN
	2 vs. Rest	0.003	0.002	0.035	0.002	0.007	NaN	NaN
	3 vs. Rest	0.006	0.004	0.010	0.024	0.008	NaN	NaN
	Random	0.006	0.003	0.013	0.009	0.015	NaN	NaN
Meltome	Random	0.022	0.004	0.007	0.011	0.006	NaN	NaN

Table G: Test set ρ for models trained on OHE representation ($\uparrow$)

Dataset	Model<br>Split	Dropout	Ensemble	Evidential	MVE	SVI	GP	BRR
AAV	7 vs. Rest	0.727	0.737	0.738	0.742	0.713	NaN	0.640
	Random	0.918	0.922	0.926	0.927	0.916	NaN	0.834
	Sampled vs. Designed	0.762	0.769	0.770	0.765	0.762	NaN	0.657
GB1	1 vs. Rest	0.018	0.071	-0.081	-0.006	0.126	0.211	0.249
	2 vs. Rest	0.156	0.304	0.351	0.268	0.313	0.694	0.555
	3 vs. Rest	0.815	0.817	0.818	0.815	0.808	0.850	0.753
	Random	0.890	0.894	0.872	0.881	0.880	0.922	0.789
Meltome	Random	0.340	0.373	0.441	0.403	0.344	NaN	NaN

Table H: Test set ρ standard deviation for models trained on OHE representation

Dataset	Model<br>Split	Dropout	Ensemble	Evidential	MVE	SVI	GP	BRR
AAV	7 vs. Rest	0.004	0.001	0.002	0.001	0.002	NaN	NaN
	Random	0.000	0.000	0.000	0.000	0.001	NaN	NaN
	Sampled vs. Designed	0.007	0.002	0.002	0.004	0.006	NaN	NaN
GB1	1 vs. Rest	0.031	0.059	0.105	0.080	0.080	NaN	NaN
	2 vs. Rest	0.051	0.020	0.080	0.030	0.029	NaN	NaN
	3 vs. Rest	0.002	0.002	0.004	0.005	0.004	NaN	NaN
	Random	0.001	0.001	0.001	0.003	0.002	NaN	NaN
Meltome	Random	0.013	0.002	0.006	0.005	0.005	NaN	NaN

Table I: Test set ρ_{unc} for models trained on OHE representation (↑)

Dataset	Model Split	Dropout	Ensemble	Evidential	MVE	SVI	GP	BRR
AAV	7 vs. Rest	0.001	0.013	0.142	0.178	-0.114	NaN	0.145
	Random	-0.019	0.064	0.194	0.229	-0.030	NaN	-0.007
	Sampled vs. Designed	0.074	0.120	0.216	0.093	0.173	NaN	0.486
GB1	1 vs. Rest	-0.005	-0.059	-0.015	0.009	-0.032	0.004	0.153
	2 vs. Rest	-0.006	0.022	0.088	0.181	-0.067	-0.323	0.092
	3 vs. Rest	0.050	0.060	0.598	0.563	0.028	-0.621	-0.246
	Random	-0.016	0.085	0.718	0.752	-0.003	-0.370	-0.254
Meltome	Random	-0.097	0.053	0.267	0.331	-0.203	NaN	NaN

Table J: Test set ρ_{unc} standard deviation for models trained on OHE representation

Dataset	Model Split	Dropout	Ensemble	Evidential	MVE	SVI	GP	BRR
AAV	7 vs. Rest	0.024	0.032	0.072	0.011	0.061	NaN	NaN
	Random	0.009	0.009	0.006	0.003	0.018	NaN	NaN
	Sampled vs. Designed	0.010	0.031	0.034	0.049	0.071	NaN	NaN
GB1	1 vs. Rest	0.006	0.098	0.023	0.078	0.054	NaN	NaN
	2 vs. Rest	0.008	0.042	0.120	0.048	0.041	NaN	NaN
	3 vs. Rest	0.023	0.029	0.032	0.053	0.230	NaN	NaN
	Random	0.008	0.071	0.014	0.006	0.159	NaN	NaN
Meltome	Random	0.017	0.017	0.019	0.038	0.027	NaN	NaN

Table K: Test set % coverage for models trained on OHE representation (↑)

Dataset	Model Split	Dropout	Ensemble	Evidential	MVE	SVI	GP	BRR
AAV	7 vs. Rest	0.519	0.354	1.000	0.276	0.198	NaN	0.767
	Random	0.641	0.356	1.000	0.438	0.268	NaN	0.955
	Sampled vs. Designed	0.400	0.264	1.000	0.161	0.154	NaN	0.805
GB1	1 vs. Rest	0.025	0.010	0.795	0.372	0.020	0.942	0.026
	2 vs. Rest	0.066	0.024	0.990	0.907	0.050	0.884	0.783
	3 vs. Rest	0.412	0.182	1.000	0.440	0.185	0.933	0.902
	Random	0.727	0.392	0.999	0.431	0.378	0.977	0.959
Meltome	Random	0.554	0.457	1.000	0.494	0.277	NaN	NaN

Table L: Test set % coverage standard deviation for models trained on OHE representation

Dataset	Model Split	Dropout	Ensemble	Evidential	MVE	SVI	GP	BRR
AAV	7 vs. Rest	0.020	0.029	0.000	0.020	0.066	NaN	NaN
	Random	0.023	0.055	0.000	0.032	0.062	NaN	NaN
	Sampled vs. Designed	0.020	0.031	0.000	0.023	0.033	NaN	NaN
GB1	1 vs. Rest	0.002	0.006	0.008	0.024	0.002	NaN	NaN
	2 vs. Rest	0.002	0.010	0.007	0.002	0.007	NaN	NaN
	3 vs. Rest	0.013	0.023	0.000	0.018	0.031	NaN	NaN
	Random	0.016	0.063	0.000	0.023	0.093	NaN	NaN
Meltome	Random	0.048	0.044	0.000	0.096	0.032	NaN	NaN

Table M: Test set $4\sigma/R$ for models trained on OHE representation (↓)

Dataset	Model Split	Dropout	Ensemble	Evidential	MVE	SVI	GP	BRR
AAV	7 vs. Rest	0.006	0.003	0.115	0.003	0.002	NaN	0.438
	Random	0.006	0.003	0.132	0.003	0.002	NaN	0.184
	Sampled vs. Designed	0.006	0.003	0.103	0.002	0.002	NaN	0.191
GB1	1 vs. Rest	0.063	0.026	1.791	0.881	0.049	0.274	0.015
	2 vs. Rest	0.010	0.003	0.336	0.137	0.007	0.116	0.107
	3 vs. Rest	0.018	0.008	0.457	0.030	0.007	0.098	0.099
	Random	0.015	0.005	0.332	0.016	0.006	0.052	0.069
Meltome	Random	0.003	0.002	0.098	0.003	0.001	NaN	NaN

Table N: Test set $4\sigma/R$ standard deviation for models trained on OHE representation

Dataset	Model Split	Dropout	Ensemble	Evidential	MVE	SVI	GP	BRR
AAV	7 vs. Rest	0.000	0.000	0.008	0.000	0.001	NaN	NaN
	Random	0.000	0.000	0.003	0.000	0.000	NaN	NaN
	Sampled vs. Designed	0.000	0.000	0.013	0.000	0.000	NaN	NaN
GB1	1 vs. Rest	0.007	0.013	0.116	0.106	0.004	NaN	NaN
	2 vs. Rest	0.000	0.001	0.058	0.002	0.001	NaN	NaN
	3 vs. Rest	0.001	0.001	0.011	0.001	0.001	NaN	NaN
	Random	0.001	0.001	0.041	0.001	0.001	NaN	NaN
Meltome	Random	0.000	0.000	0.011	0.001	0.000	NaN	NaN

Table O: Test set miscalibration area for models trained on OHE representation (↓)

Dataset	Model Split	Dropout	Ensemble	Evidential	MVE	SVI	GP	BRR
AAV	7 vs. Rest	0.281	0.355	0.404	0.387	0.421	NaN	0.102
	Random	0.215	0.354	0.445	0.318	0.392	NaN	0.004
	Sampled vs. Designed	0.336	0.393	0.373	0.435	0.438	NaN	0.095
GB1	1 vs. Rest	0.489	0.495	0.218	0.357	0.492	0.198	0.488
	2 vs. Rest	0.473	0.490	0.188	0.124	0.479	0.078	0.137
	3 vs. Rest	0.332	0.425	0.402	0.324	0.426	0.122	0.045
	Random	0.125	0.329	0.432	0.317	0.342	0.186	0.038
Meltome	Random	0.263	0.306	0.450	0.289	0.387	NaN	NaN

Table P: Test set miscalibration area standard deviation for models trained on OHE representation

Dataset	Model Split	Dropout	Ensemble	Evidential	MVE	SVI	GP	BRR
AAV	7 vs. Rest	0.009	0.012	0.007	0.008	0.028	NaN	NaN
	Random	0.013	0.023	0.001	0.015	0.026	NaN	NaN
	Sampled vs. Designed	0.009	0.014	0.015	0.009	0.013	NaN	NaN
GB1	1 vs. Rest	0.001	0.002	0.019	0.017	0.001	NaN	NaN
	2 vs. Rest	0.001	0.004	0.046	0.006	0.003	NaN	NaN
	3 vs. Rest	0.010	0.009	0.003	0.009	0.012	NaN	NaN
	Random	0.013	0.030	0.007	0.012	0.040	NaN	NaN
Meltome	Random	0.027	0.020	0.007	0.044	0.016	NaN	NaN

Table Q: Test set $\overline{NLL}$ for models trained on OHE representation (↓)

Dataset	Model Split	Dropout	Ensemble	Evidential	MVE	SVI	GP	BRR
AAV	7 vs. Rest	5.427	18.148	3.342	26.312	51.394	NaN	3.667
	Random	3.678	19.773	3.430	7.856	24.652	NaN	2.058
	Sampled vs. Designed	9.132	31.511	3.266	64.200	62.343	NaN	3.183
GB1	1 vs. Rest	2977.270	23918.764	3.259	12.176	3946.655	1.937	9871.345
	2 vs. Rest	251.599	2695.786	1.834	1.790	380.500	1.596	2.076
	3 vs. Rest	12.551	96.398	2.294	56.250	74.424	1.216	1.488
	Random	2.413	29.654	2.165	90.282	24.882	0.757	1.265
Meltome	Random	7.764	11.874	5.580	28.920	27.409	NaN	NaN

Table R: Test set $\overline{NLL}$ standard deviation for models trained on OHE representation

Dataset	Model Split	Dropout	Ensemble	Evidential	MVE	SVI	GP	BRR
AAV	7 vs. Rest	0.387	4.295	0.068	4.196	40.809	NaN	NaN
	Random	0.318	6.819	0.020	1.294	11.128	NaN	NaN
	Sampled vs. Designed	0.883	7.237	0.113	13.766	25.906	NaN	NaN
GB1	1 vs. Rest	695.600	29378.989	0.298	2.868	606.968	NaN	NaN
	2 vs. Rest	17.640	2357.116	0.107	0.017	89.908	NaN	NaN
	3 vs. Rest	0.858	36.493	0.035	26.101	25.438	NaN	NaN
	Random	0.058	19.352	0.145	6.183	12.162	NaN	NaN
Meltome	Random	1.351	2.203	0.122	14.851	7.230	NaN	NaN

Table S: Test set $\overline{NLL_{opt}}$ for models trained on OHE representation

Dataset	Model / Split	Dropout	Ensemble	Evidential	MVE	SVI	GP	BRR
AAV	7 vs. Rest	1.237	1.202	1.282	1.214	1.277	NaN	2.183
	Random	0.899	0.868	0.797	0.805	0.991	NaN	1.408
	Sampled vs. Designed	1.538	1.498	1.481	1.517	1.542	NaN	1.834
GB1	1 vs. Rest	0.878	0.879	0.855	0.881	0.883	1.293	1.483
	2 vs. Rest	1.055	1.049	0.908	1.033	1.042	0.418	0.977
	3 vs. Rest	0.438	0.414	0.269	0.308	0.433	0.037	0.730
	Random	-0.079	-0.180	-0.223	-0.451	0.025	-0.374	0.541
Meltome	Random	2.929	2.851	2.820	2.875	2.891	NaN	NaN

Table T: Test set $\overline{NLL_{opt}}$ standard deviation for models trained on OHE representation

Dataset	Model / Split	Dropout	Ensemble	Evidential	MVE	SVI	GP	BRR
AAV	7 vs. Rest	0.030	0.003	0.029	0.025	0.095	NaN	NaN
	Random	0.012	0.008	0.012	0.017	0.135	NaN	NaN
	Sampled vs. Designed	0.024	0.010	0.038	0.031	0.018	NaN	NaN
GB1	1 vs. Rest	0.006	0.006	0.014	0.003	0.010	NaN	NaN
	2 vs. Rest	0.005	0.004	0.029	0.007	0.021	NaN	NaN
	3 vs. Rest	0.025	0.011	0.047	0.064	0.034	NaN	NaN
	Random	0.036	0.024	0.065	0.063	0.102	NaN	NaN
Meltome	Random	0.026	0.008	0.010	0.011	0.011	NaN	NaN

Table U: Test set $\overline{NLL}$ / $\overline{NLL_{opt}}$ ratio for models trained on OHE representation (↓)

Dataset	Model / Split	Dropout	Ensemble	Evidential	MVE	SVI	GP	BRR
AAV	7 vs. Rest	4.387	15.102	2.608	21.684	38.925	NaN	1.680
	Random	4.089	22.765	4.305	9.750	24.970	NaN	1.462
	Sampled vs. Designed	5.939	21.014	2.206	42.262	40.329	NaN	1.736
GB1	1 vs. Rest	3390.230	27281.195	3.810	13.818	4474.286	1.498	6656.782
	2 vs. Rest	238.554	2564.788	2.024	1.732	365.159	3.816	2.125
	3 vs. Rest	28.691	232.149	8.737	175.004	173.197	33.170	2.038
	Random	-49.594	-161.397	-10.754	-204.256	220.658	-2.026	2.339
Meltome	Random	2.652	4.164	1.979	10.050	9.483	NaN	NaN

Table V: Test set $\overline{NLL}$ / $\overline{NLL_{opt}}$ ratio standard deviation for models trained on OHE representation

Dataset	Model / Split	Dropout	Ensemble	Evidential	MVE	SVI	GP	BRR
AAV	7 vs. Rest	0.272	3.586	0.063	3.527	27.500	NaN	NaN
	Random	0.323	7.775	0.072	1.512	11.698	NaN	NaN
	Sampled vs. Designed	0.576	4.703	0.098	8.719	16.374	NaN	NaN
GB1	1 vs. Rest	791.103	33631.622	0.305	3.239	703.321	NaN	NaN
	2 vs. Rest	16.865	2230.032	0.180	0.010	85.957	NaN	NaN
	3 vs. Rest	1.659	85.797	1.520	46.328	64.975	NaN	NaN
	Random	53.151	87.677	4.505	39.077	592.369	NaN	NaN
Meltome	Random	0.472	0.765	0.044	5.158	2.520	NaN	NaN

Table W: Test set RMSE for models trained on ESM representation (↓)

Dataset	Model / Split	Dropout	Ensemble	Evidential	MVE	SVI	GP	BRR
AAV	7 vs. Rest	2.241	2.213	2.157	2.168	2.217	NaN	4.083
	Random	1.875	1.734	1.719	1.793	1.855	NaN	1.582
	Sampled vs. Designed	2.648	2.555	2.581	2.639	2.649	NaN	2.942
GB1	1 vs. Rest	1.333	1.314	1.306	1.328	1.383	1.360	2.413
	2 vs. Rest	1.127	1.111	1.086	1.118	1.140	1.111	1.484
	3 vs. Rest	0.918	0.885	0.908	0.911	0.930	0.920	0.949
	Random	0.761	0.719	0.812	0.775	0.780	0.669	0.631
Meltome	Random	6.722	6.536	6.598	6.764	6.713	6.447	6.936

Table X: Test set RMSE standard deviation for models trained on ESM representation

Dataset	Model<br>Split	Dropout	Ensemble	Evidential	MVE	SVI	GP	BRR
AAV	7 vs. Rest	0.076	0.098	0.103	0.116	0.143	NaN	NaN
	Random	0.111	0.048	0.103	0.143	0.109	NaN	NaN
	Sampled vs. Designed	0.089	0.040	0.086	0.118	0.108	NaN	NaN
GB1	1 vs. Rest	0.051	0.026	0.044	0.050	0.058	NaN	NaN
	2 vs. Rest	0.018	0.007	0.016	0.018	0.012	NaN	NaN
	3 vs. Rest	0.036	0.015	0.023	0.031	0.033	NaN	NaN
	Random	0.047	0.021	0.044	0.045	0.038	NaN	NaN
Meltome	Random	0.156	0.047	0.137	0.157	0.160	NaN	NaN

Table Y: Test set MAE for models trained on ESM representation (↓)

Dataset	Model<br>Split	Dropout	Ensemble	Evidential	MVE	SVI	GP	BRR
AAV	7 vs. Rest	1.844	1.822	1.738	1.769	1.819	NaN	2.798
	Random	1.474	1.354	1.306	1.400	1.458	NaN	1.235
	Sampled vs. Designed	2.218	2.140	2.137	2.199	2.203	NaN	2.044
GB1	1 vs. Rest	1.143	1.127	1.110	1.137	1.188	1.173	1.949
	2 vs. Rest	0.868	0.858	0.819	0.862	0.886	0.845	1.123
	3 vs. Rest	0.687	0.656	0.627	0.654	0.697	0.680	0.731
	Random	0.537	0.502	0.513	0.515	0.556	0.471	0.478
Meltome	Random	5.024	4.862	4.806	4.974	4.999	4.777	5.218

Table Z: Test set MAE standard deviation for models trained on ESM representation

Dataset	Model<br>Split	Dropout	Ensemble	Evidential	MVE	SVI	GP	BRR
AAV	7 vs. Rest	0.062	0.087	0.091	0.103	0.129	NaN	NaN
	Random	0.102	0.046	0.083	0.127	0.098	NaN	NaN
	Sampled vs. Designed	0.097	0.043	0.092	0.100	0.111	NaN	NaN
GB1	1 vs. Rest	0.060	0.031	0.056	0.059	0.058	NaN	NaN
	2 vs. Rest	0.020	0.008	0.025	0.022	0.013	NaN	NaN
	3 vs. Rest	0.039	0.014	0.024	0.036	0.039	NaN	NaN
	Random	0.039	0.018	0.034	0.040	0.033	NaN	NaN
Meltome	Random	0.119	0.058	0.103	0.125	0.126	NaN	NaN

Table AA: Test set R^2 for models trained on ESM representation (↑)

Dataset	Model<br>Split	Dropout	Ensemble	Evidential	MVE	SVI	GP	BRR
AAV	7 vs. Rest	-0.216	-0.187	-0.128	-0.140	-0.193	NaN	-3.034
	Random	0.628	0.683	0.687	0.659	0.636	NaN	0.736
	Sampled vs. Designed	0.418	0.459	0.447	0.422	0.418	NaN	0.282
GB1	1 vs. Rest	-0.191	-0.157	-0.144	-0.183	-0.284	-0.240	-2.900
	2 vs. Rest	0.162	0.185	0.222	0.174	0.142	0.185	-0.455
	3 vs. Rest	0.481	0.518	0.493	0.489	0.467	0.480	0.446
	Random	0.600	0.644	0.546	0.585	0.580	0.692	0.726
Meltome	Random	0.665	0.684	0.678	0.661	0.666	0.692	0.644

Table AB: Test set R^2 standard deviation for models trained on ESM representation

Dataset	Model<br>Split	Dropout	Ensemble	Evidential	MVE	SVI	GP	BRR
AAV	7 vs. Rest	0.081	0.105	0.108	0.121	0.150	NaN	NaN
	Random	0.043	0.018	0.037	0.054	0.043	NaN	NaN
	Sampled vs. Designed	0.039	0.017	0.037	0.051	0.047	NaN	NaN
GB1	1 vs. Rest	0.090	0.045	0.076	0.088	0.106	NaN	NaN
	2 vs. Rest	0.027	0.010	0.022	0.026	0.019	NaN	NaN
	3 vs. Rest	0.040	0.017	0.026	0.035	0.037	NaN	NaN
	Random	0.048	0.021	0.049	0.047	0.040	NaN	NaN
Meltome	Random	0.016	0.005	0.013	0.016	0.016	NaN	NaN

Table AC: Test set ρ for models trained on ESM representation ($\uparrow$)

Dataset	Model / Split	Dropout	Ensemble	Evidential	MVE	SVI	GP	BRR
AAV	7 vs. Rest	0.522	0.557	0.557	0.542	0.531	NaN	0.586
	Random	0.816	0.842	0.837	0.826	0.813	NaN	0.876
	Sampled vs. Designed	0.682	0.714	0.701	0.692	0.681	NaN	0.696
GB1	1 vs. Rest	0.202	0.303	0.222	0.200	0.152	0.315	0.279
	2 vs. Rest	0.509	0.535	0.552	0.530	0.490	0.523	0.528
	3 vs. Rest	0.769	0.788	0.790	0.776	0.762	0.779	0.809
	Random	0.822	0.842	0.837	0.832	0.813	0.861	0.871
Meltome	Random	0.638	0.664	0.668	0.654	0.633	0.650	0.591

Table AD: Test set ρ standard deviation for models trained on ESM representation

Dataset	Model / Split	Dropout	Ensemble	Evidential	MVE	SVI	GP	BRR
AAV	7 vs. Rest	0.028	0.011	0.023	0.034	0.020	NaN	NaN
	Random	0.026	0.008	0.023	0.030	0.024	NaN	NaN
	Sampled vs. Designed	0.019	0.010	0.020	0.037	0.019	NaN	NaN
GB1	1 vs. Rest	0.129	0.044	0.142	0.152	0.201	NaN	NaN
	2 vs. Rest	0.022	0.009	0.010	0.018	0.019	NaN	NaN
	3 vs. Rest	0.013	0.006	0.012	0.016	0.010	NaN	NaN
	Random	0.020	0.009	0.017	0.019	0.016	NaN	NaN
Meltome	Random	0.025	0.006	0.011	0.021	0.024	NaN	NaN

Table AE: Test set ρ_{unc} for models trained on ESM representation ($\uparrow$)

Dataset	Model / Split	Dropout	Ensemble	Evidential	MVE	SVI	GP	BRR
AAV	7 vs. Rest	-0.235	0.129	-0.069	0.084	-0.162	NaN	0.441
	Random	-0.194	0.143	-0.016	0.274	-0.110	NaN	0.008
	Sampled vs. Designed	-0.142	0.075	0.206	0.005	-0.066	NaN	0.349
GB1	1 vs. Rest	0.136	0.099	-0.057	0.099	0.166	0.233	0.087
	2 vs. Rest	0.037	0.084	0.323	0.358	0.018	0.068	0.245
	3 vs. Rest	-0.033	0.043	0.501	0.554	-0.027	-0.237	0.056
	Random	0.054	0.223	0.621	0.647	0.145	-0.347	-0.072
Meltome	Random	0.214	0.241	0.327	0.376	0.275	0.109	0.119

Table AF: Test set ρ_{unc} standard deviation for models trained on ESM representation

Dataset	Model / Split	Dropout	Ensemble	Evidential	MVE	SVI	GP	BRR
AAV	7 vs. Rest	0.036	0.064	0.027	0.040	0.123	NaN	NaN
	Random	0.046	0.028	0.100	0.016	0.045	NaN	NaN
	Sampled vs. Designed	0.027	0.040	0.025	0.047	0.203	NaN	NaN
GB1	1 vs. Rest	0.047	0.028	0.081	0.118	0.045	NaN	NaN
	2 vs. Rest	0.025	0.056	0.035	0.054	0.045	NaN	NaN
	3 vs. Rest	0.069	0.093	0.020	0.021	0.141	NaN	NaN
	Random	0.031	0.072	0.044	0.041	0.214	NaN	NaN
Meltome	Random	0.031	0.014	0.013	0.008	0.012	NaN	NaN

Table AG: Test set % coverage for models trained on ESM representation ($\uparrow$)

Dataset	Model / Split	Dropout	Ensemble	Evidential	MVE	SVI	GP	BRR
AAV	7 vs. Rest	0.082	0.289	1.000	0.499	0.052	NaN	0.721
	Random	0.110	0.352	1.000	0.759	0.047	NaN	0.950
	Sampled vs. Designed	0.064	0.195	1.000	0.378	0.039	NaN	0.817
GB1	1 vs. Rest	0.104	0.126	0.985	0.639	0.079	0.943	0.012
	2 vs. Rest	0.146	0.183	0.798	0.264	0.099	0.913	0.801
	3 vs. Rest	0.127	0.289	0.998	0.571	0.076	0.935	0.890
	Random	0.161	0.377	1.000	0.586	0.102	0.968	0.947
Meltome	Random	0.097	0.283	1.000	0.711	0.059	0.956	0.932

Table AH: Test set % coverage standard deviation for models trained on ESM representation

Dataset	Model<br>Split	Dropout	Ensemble	Evidential	MVE	SVI	GP	BRR
AAV	7 vs. Rest	0.016	0.022	0.000	0.079	0.040	NaN	NaN
	Random	0.037	0.050	0.000	0.042	0.021	NaN	NaN
	Sampled vs. Designed	0.017	0.019	0.000	0.043	0.033	NaN	NaN
GB1	1 vs. Rest	0.030	0.064	0.002	0.033	0.017	NaN	NaN
	2 vs. Rest	0.054	0.024	0.123	0.124	0.044	NaN	NaN
	3 vs. Rest	0.046	0.024	0.002	0.069	0.041	NaN	NaN
	Random	0.072	0.041	0.000	0.078	0.065	NaN	NaN
Meltome	Random	0.031	0.017	0.000	0.047	0.021	NaN	NaN

Table AI: Test set $4\sigma/R$ for models trained on ESM representation (↓)

Dataset	Model<br>Split	Dropout	Ensemble	Evidential	MVE	SVI	GP	BRR
AAV	7 vs. Rest	0.001	0.005	2.989	0.008	0.001	NaN	0.162
	Random	0.001	0.004	0.157	0.011	0.000	NaN	0.155
	Sampled vs. Designed	0.001	0.004	0.425	0.008	0.001	NaN	0.152
GB1	1 vs. Rest	0.054	0.062	0.555	0.254	0.042	0.275	0.008
	2 vs. Rest	0.013	0.018	0.127	0.031	0.009	0.120	0.124
	3 vs. Rest	0.006	0.013	0.271	0.042	0.003	0.102	0.088
	Random	0.002	0.006	0.148	0.019	0.002	0.063	0.050
Meltome	Random	0.000	0.001	0.030	0.003	0.000	0.605	0.580

Table AJ: Test set $4\sigma/R$ standard deviation for models trained on ESM representation

Dataset	Model<br>Split	Dropout	Ensemble	Evidential	MVE	SVI	GP	BRR
AAV	7 vs. Rest	0.000	0.000	1.828	0.001	0.001	NaN	NaN
	Random	0.000	0.001	0.015	0.002	0.000	NaN	NaN
	Sampled vs. Designed	0.000	0.000	0.185	0.001	0.001	NaN	NaN
GB1	1 vs. Rest	0.014	0.030	0.020	0.019	0.011	NaN	NaN
	2 vs. Rest	0.004	0.003	0.045	0.012	0.004	NaN	NaN
	3 vs. Rest	0.002	0.002	0.085	0.004	0.001	NaN	NaN
	Random	0.001	0.001	0.052	0.002	0.001	NaN	NaN
Meltome	Random	0.000	0.000	0.010	0.000	0.000	NaN	NaN

Table AK: Test set miscalibration area for models trained on ESM representation (↓)

Dataset	Model<br>Split	Dropout	Ensemble	Evidential	MVE	SVI	GP	BRR
AAV	7 vs. Rest	0.467	0.387	0.457	0.297	0.479	NaN	0.173
	Random	0.455	0.356	0.392	0.162	0.481	NaN	0.016
	Sampled vs. Designed	0.474	0.423	0.414	0.348	0.484	NaN	0.107
GB1	1 vs. Rest	0.459	0.451	0.053	0.268	0.469	0.202	0.494
	2 vs. Rest	0.442	0.427	0.107	0.397	0.461	0.057	0.121
	3 vs. Rest	0.449	0.379	0.293	0.273	0.469	0.022	0.066
	Random	0.434	0.340	0.345	0.252	0.461	0.123	0.037
Meltome	Random	0.460	0.384	0.383	0.190	0.476	0.055	0.019

Table AL: Test set miscalibration area standard deviation for models trained on ESM representation

Dataset	Model<br>Split	Dropout	Ensemble	Evidential	MVE	SVI	GP	BRR
AAV	7 vs. Rest	0.007	0.009	0.006	0.034	0.016	NaN	NaN
	Random	0.015	0.021	0.036	0.022	0.008	NaN	NaN
	Sampled vs. Designed	0.007	0.007	0.017	0.018	0.013	NaN	NaN
GB1	1 vs. Rest	0.013	0.024	0.011	0.010	0.007	NaN	NaN
	2 vs. Rest	0.021	0.010	0.088	0.049	0.018	NaN	NaN
	3 vs. Rest	0.018	0.011	0.054	0.027	0.017	NaN	NaN
	Random	0.031	0.018	0.050	0.030	0.024	NaN	NaN
Meltome	Random	0.012	0.007	0.040	0.027	0.009	NaN	NaN

Table AM: Test set $\overline{NLL}$ for models trained on ESM representation (↓)

Dataset	Model Split	Dropout	Ensemble	Evidential	MVE	SVI	GP	BRR
AAV	7 vs. Rest	453.943	19.534	5.207	10.936	1370.946	NaN	3.667
	Random	320.970	16.868	3.338	2.488	1212.551	NaN	1.877
	Sampled vs. Designed	444.501	44.511	4.052	9.440	1913.293	NaN	2.724
GB1	1 vs. Rest	64.584	77.056	1.710	2.402	99.813	1.948	25929.804
	2 vs. Rest	138.280	76.581	7.136	599.876	293.891	1.595	2.139
	3 vs. Rest	273.534	43.638	1.726	39.261	671.193	1.348	1.468
	Random	247.572	20.924	1.647	19.394	565.041	1.053	0.966
Meltome	Random	301.052	30.996	4.423	4.672	703.508	3.282	3.360

Table AN: Test set $\overline{NLL}$ standard deviation for models trained on ESM representation

Dataset	Model Split	Dropout	Ensemble	Evidential	MVE	SVI	GP	BRR
AAV	7 vs. Rest	212.351	2.637	0.417	6.772	1189.204	NaN	NaN
	Random	202.370	6.301	0.235	0.159	816.441	NaN	NaN
	Sampled vs. Designed	209.798	7.835	0.130	1.673	1638.333	NaN	NaN
GB1	1 vs. Rest	35.213	47.869	0.030	0.105	49.676	NaN	NaN
	2 vs. Rest	98.241	21.616	9.905	894.419	240.166	NaN	NaN
	3 vs. Rest	185.984	13.701	0.219	21.462	520.042	NaN	NaN
	Random	189.300	5.493	0.213	8.673	446.637	NaN	NaN
Meltome	Random	180.252	3.816	0.334	0.447	510.500	NaN	NaN

Table AO: Test set $\overline{NLL_{opt}}$ for models trained on ESM representation

Dataset	Model Split	Dropout	Ensemble	Evidential	MVE	SVI	GP	BRR
AAV	7 vs. Rest	1.664	1.653	1.567	1.608	1.644	NaN	1.911
	Random	1.367	1.278	1.205	1.310	1.359	NaN	1.188
	Sampled vs. Designed	1.871	1.838	1.812	1.855	1.851	NaN	1.639
GB1	1 vs. Rest	1.275	1.259	1.237	1.268	1.309	1.304	1.694
	2 vs. Rest	0.847	0.841	0.764	0.836	0.883	0.864	1.053
	3 vs. Rest	0.565	0.493	0.384	0.454	0.585	0.543	0.677
	Random	0.221	0.135	-0.042	0.029	0.281	0.083	0.235
Meltome	Random	2.551	2.510	2.464	2.519	2.537	2.477	2.586

Table AP: Test set $\overline{NLL_{opt}}$ standard deviation for models trained on ESM representation

Dataset	Model Split	Dropout	Ensemble	Evidential	MVE	SVI	GP	BRR
AAV	7 vs. Rest	0.038	0.050	0.059	0.065	0.085	NaN	NaN
	Random	0.086	0.041	0.067	0.103	0.074	NaN	NaN
	Sampled vs. Designed	0.062	0.027	0.056	0.047	0.070	NaN	NaN
GB1	1 vs. Rest	0.065	0.033	0.069	0.066	0.052	NaN	NaN
	2 vs. Rest	0.039	0.014	0.057	0.041	0.025	NaN	NaN
	3 vs. Rest	0.085	0.028	0.069	0.095	0.091	NaN	NaN
	Random	0.103	0.040	0.132	0.151	0.088	NaN	NaN
Meltome	Random	0.026	0.022	0.031	0.032	0.023	NaN	NaN

Table AQ: Test set $\overline{NLL}$ / $\overline{NLL_{opt}}$ ratio for models trained on ESM representation (↓)

Dataset	Model Split	Dropout	Ensemble	Evidential	MVE	SVI	GP	BRR
AAV	7 vs. Rest	273.638	11.806	3.324	6.734	826.919	NaN	1.919
	Random	228.987	13.229	2.786	1.915	868.280	NaN	1.579
	Sampled vs. Designed	235.180	24.210	2.239	5.094	1013.232	NaN	1.662
GB1	1 vs. Rest	50.427	61.282	1.385	1.901	77.453	1.494	15310.669
	2 vs. Rest	160.595	91.310	8.750	697.394	328.277	1.846	2.031
	3 vs. Rest	453.390	89.292	4.715	90.982	1070.519	2.483	2.168
	Random	991.498	168.540	-6.076	42.706	1751.474	12.645	4.110
Meltome	Random	117.634	12.360	1.796	1.856	276.206	1.325	1.299

Table AR: Test set $\overline{NLL}$ / $\overline{NLL_{opt}}$ ratio standard deviation for models trained on ESM representation

Dataset	Model<br>Split	Dropout	Ensemble	Evidential	MVE	SVI	GP	BRR
AAV	7 vs. Rest	131.357	1.459	0.242	3.963	719.900	NaN	NaN
	Random	135.399	4.967	0.349	0.248	550.558	NaN	NaN
	Sampled vs. Designed	105.508	4.188	0.135	0.927	857.194	NaN	NaN
GB1	1 vs. Rest	26.221	37.879	0.060	0.175	41.419	NaN	NaN
	2 vs. Rest	111.562	26.618	11.442	1007.164	264.227	NaN	NaN
	3 vs. Rest	263.496	30.255	1.507	51.229	737.896	NaN	NaN
	Random	412.701	72.803	29.860	267.461	1173.469	NaN	NaN
Meltome	Random	69.806	1.609	0.152	0.186	198.660	NaN	NaN

5 Active Learning

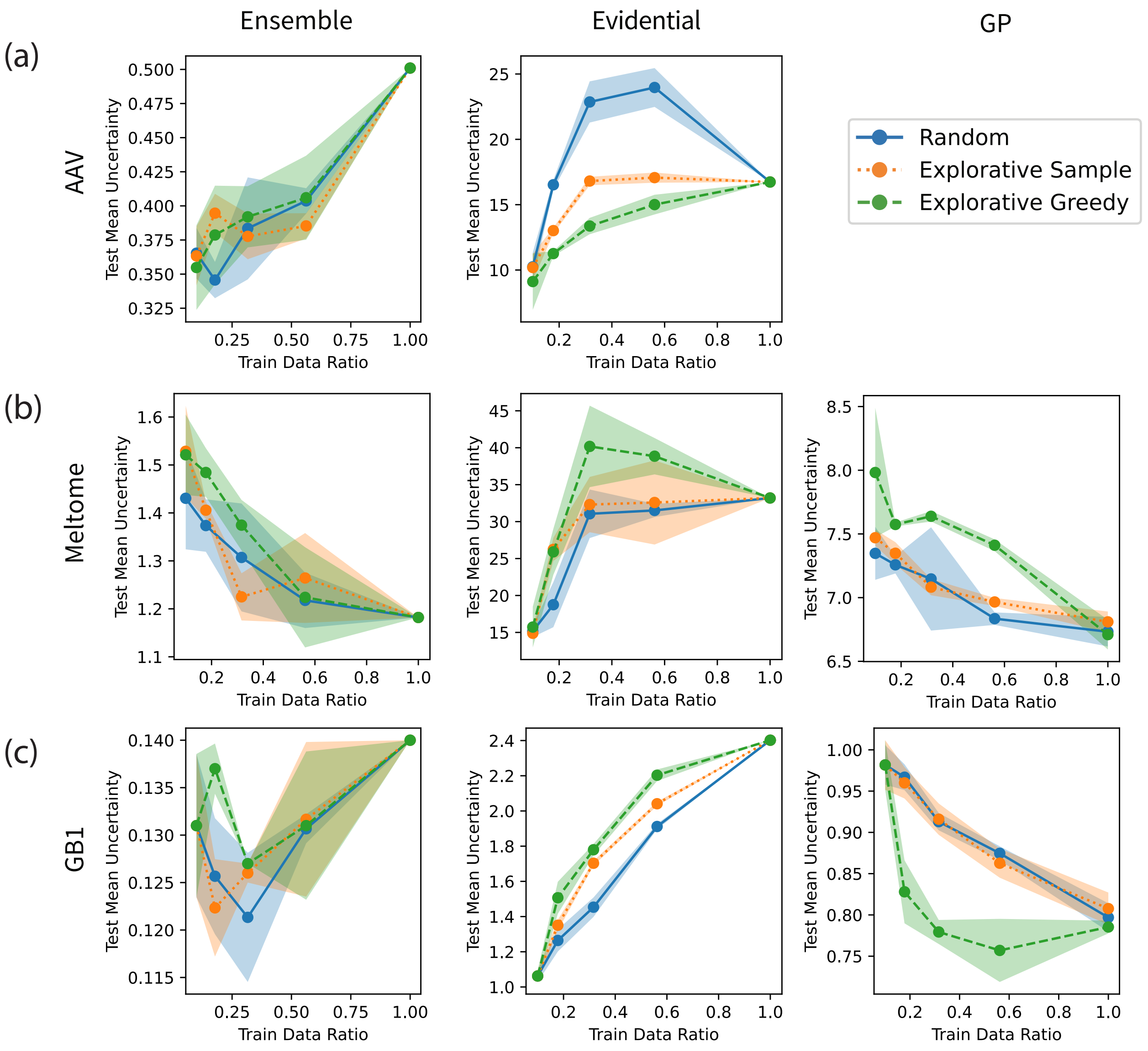

Figure E: Change in mean test set uncertainty throughout active learning for the CNN ensemble, CNN evidential, and GP methods evaluated on the AAV/Random (a), Meltome/Random (b), and GB1/Random (c) splits.

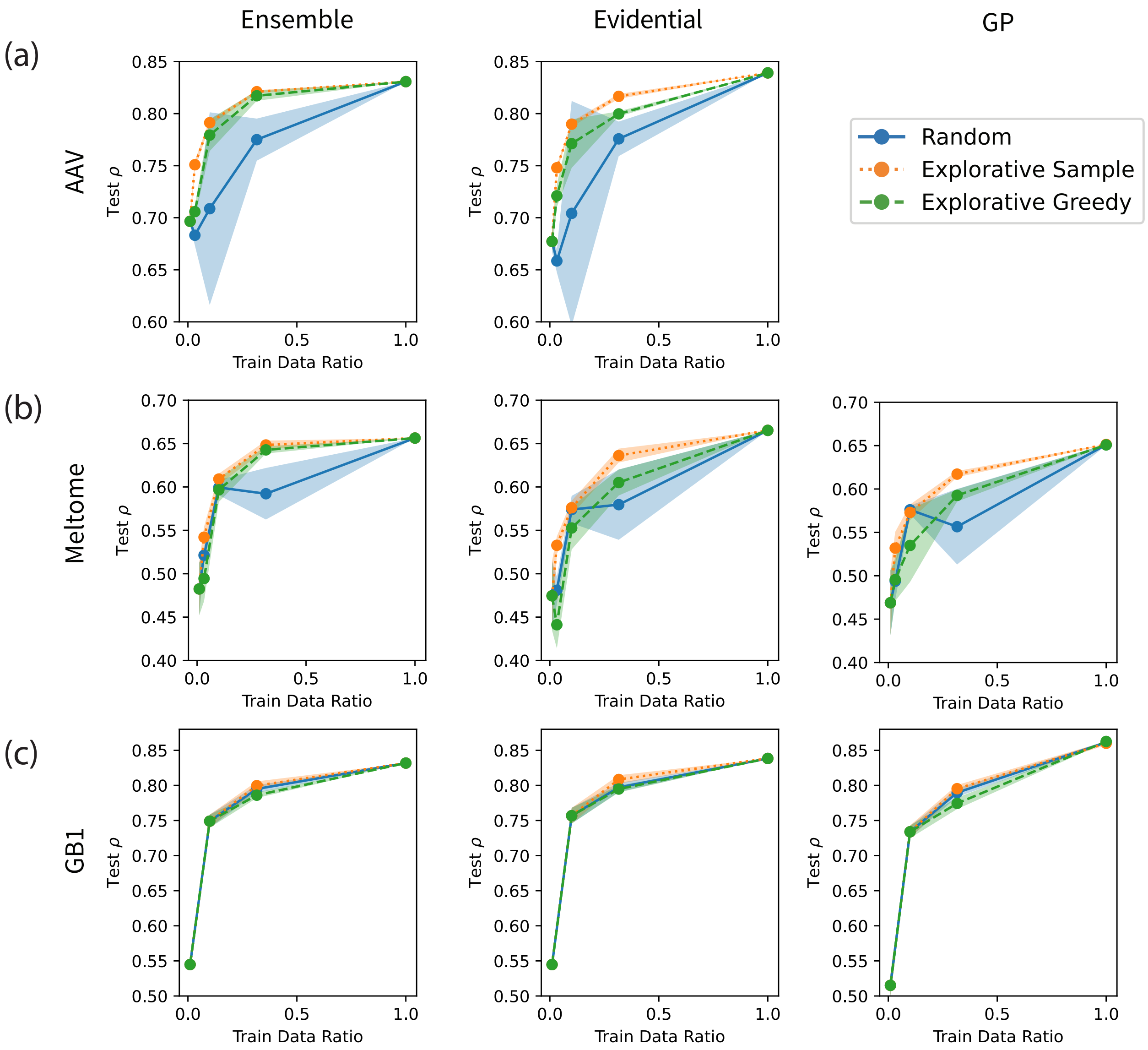

Figure F: Uncertainty-guided active learning in protein sequence-function prediction, beginning with an initial sample of 1% of the training data. Spearman rank correlation of predictions (ρ) for the CNN ensemble, CNN evidential, and GP methods evaluated on the AAV/Random (a), Meltome/Random (b), and GB1/Random (c) splits.

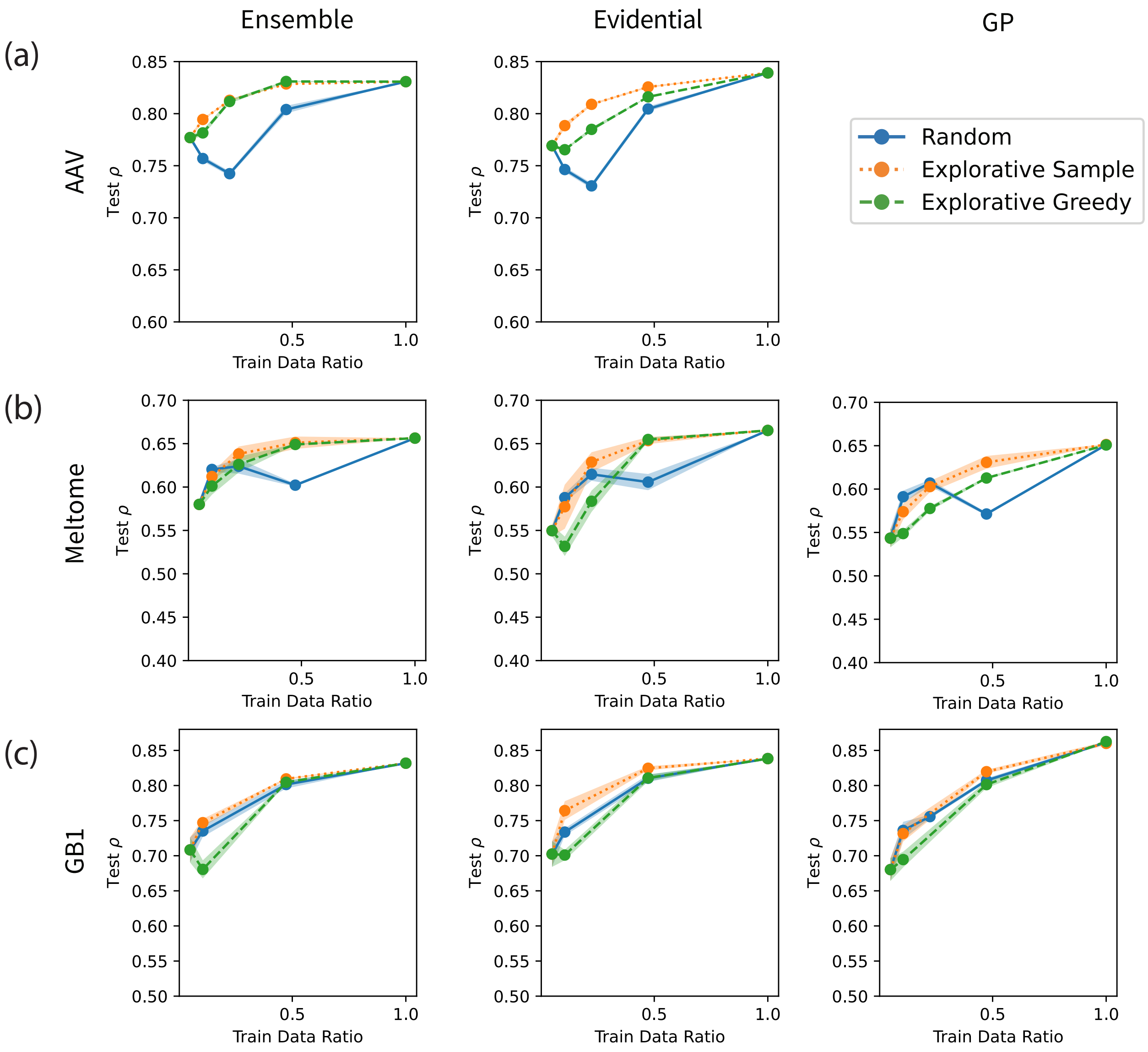

Figure G: Uncertainty-guided active learning in protein sequence-function prediction, beginning with an initial sample of 5% of the training data. Spearman rank correlation of predictions (ρ) for the CNN ensemble, CNN evidential, and GP methods evaluated on the AAV/Random (a), Meltome/Random (b), and GB1/Random (c) splits.

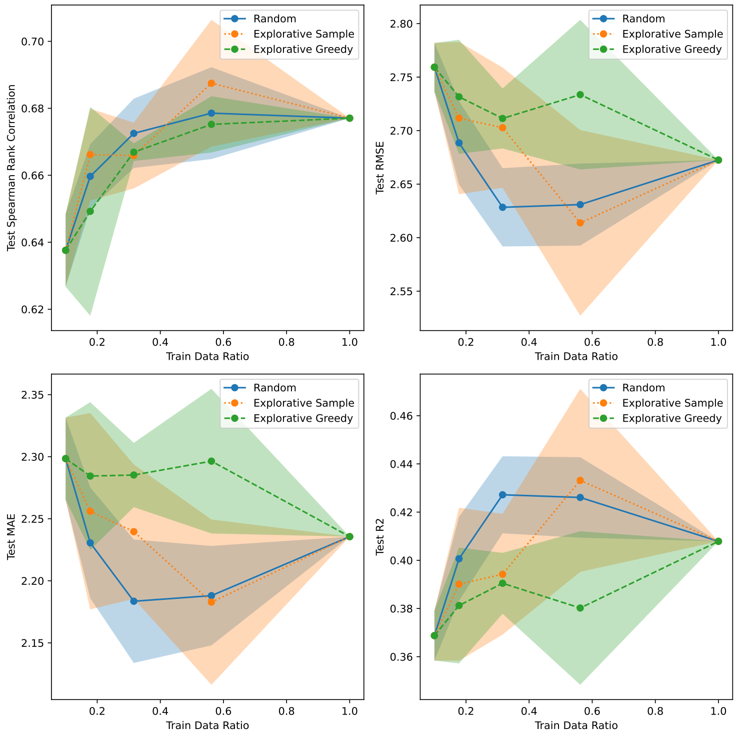

Figure H: Active learning results for AAV/Sampled vs. Designed using CNN Dropout uncertainty.

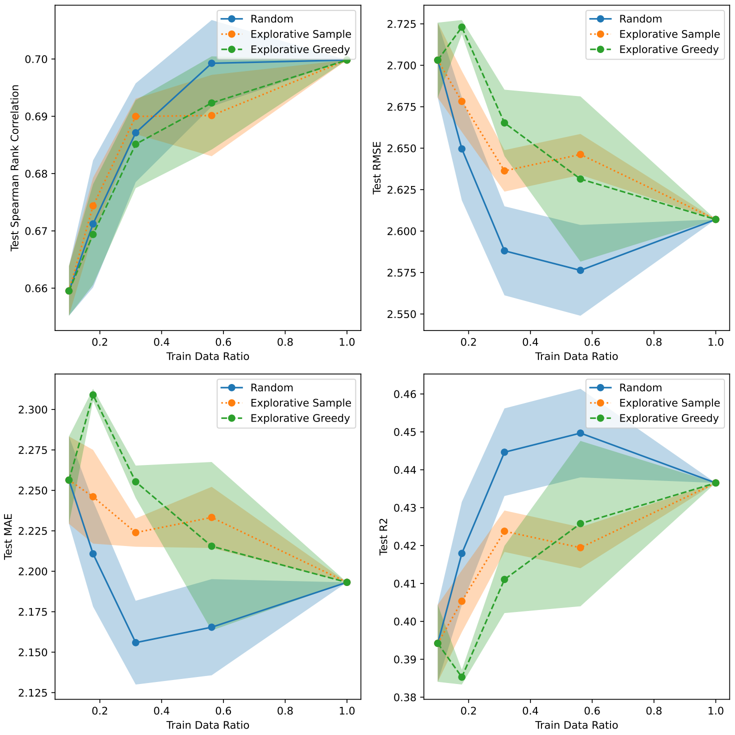

Figure I: Active learning results for AAV/Sampled vs. Designed using CNN Ensemble uncertainty.

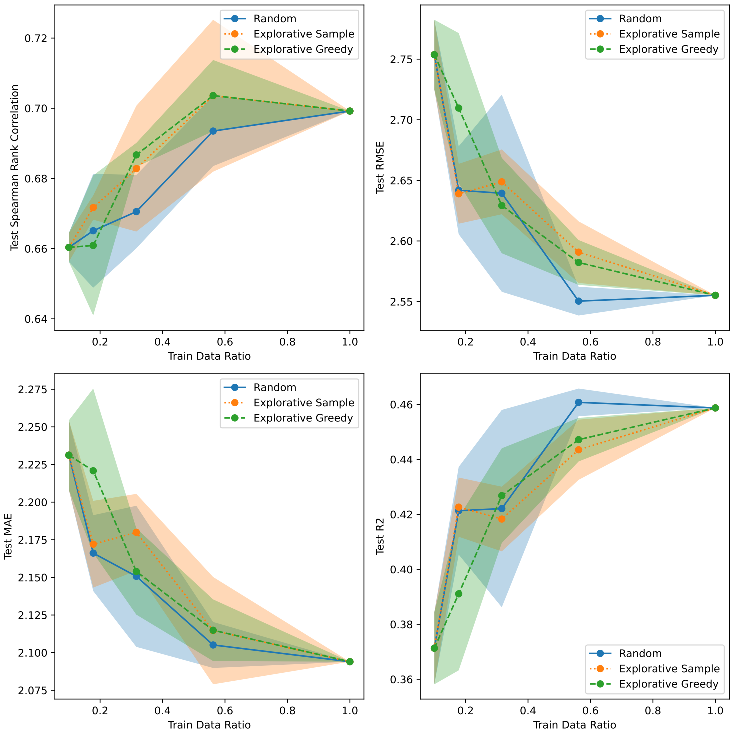

Figure J: Active learning results for AAV/Sampled vs. Designed using CNN Evidential uncertainty.

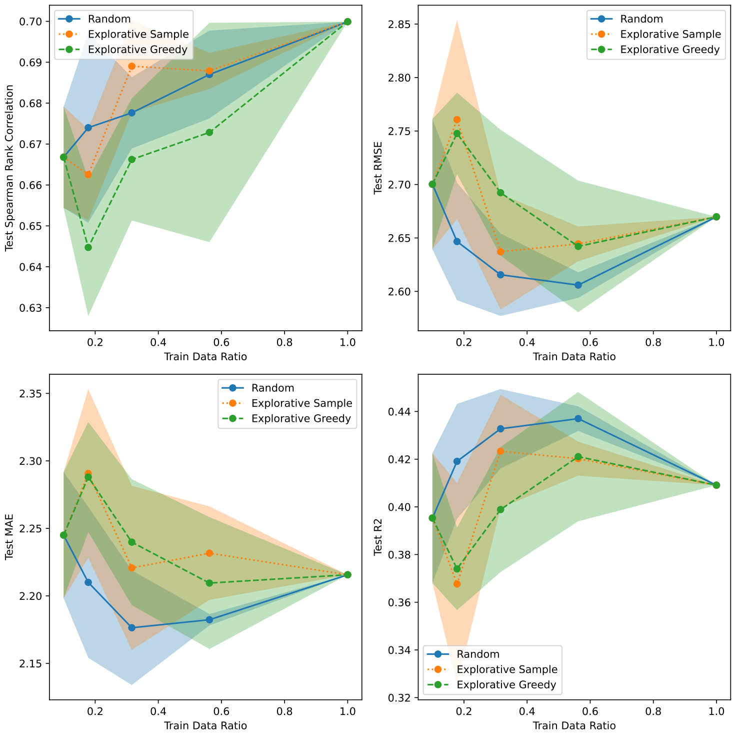

Figure K: Active learning results for AAV/Sampled vs. Designed using CNN MVE uncertainty.

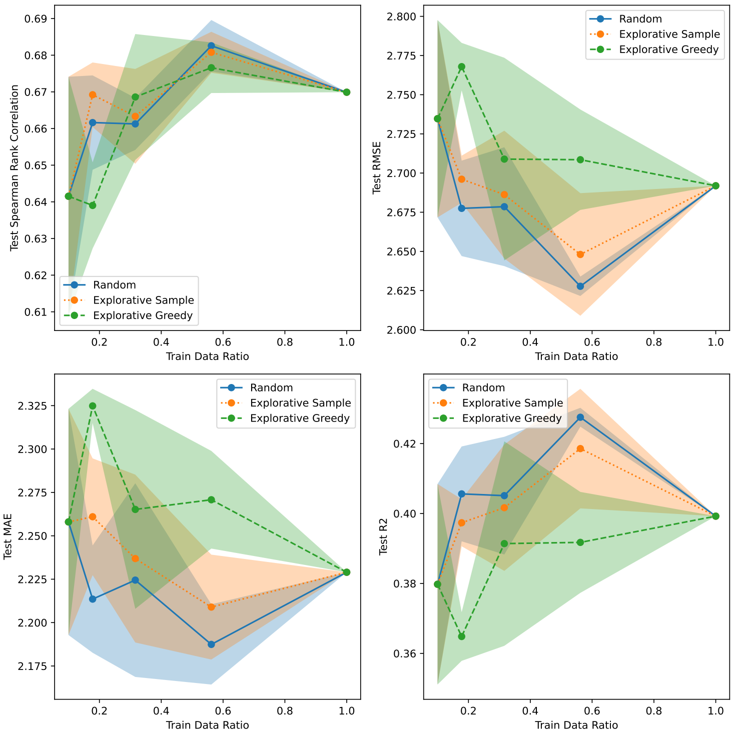

Figure L: Active learning results for AAV/Sampled vs. Designed using CNN SVI uncertainty.

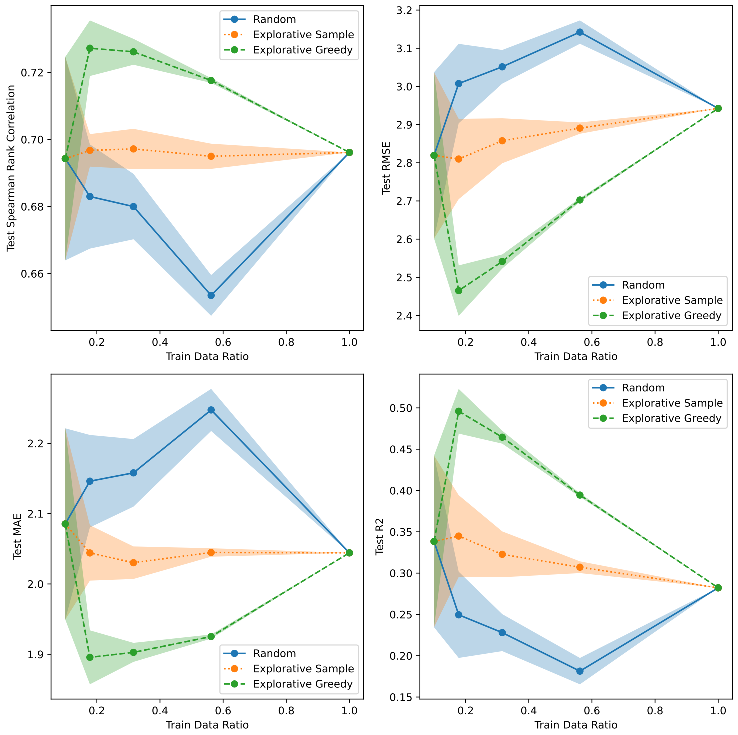

Figure M: Active learning results for AAV/Sampled vs. Designed using Linear Bayesian Ridge uncertainty.

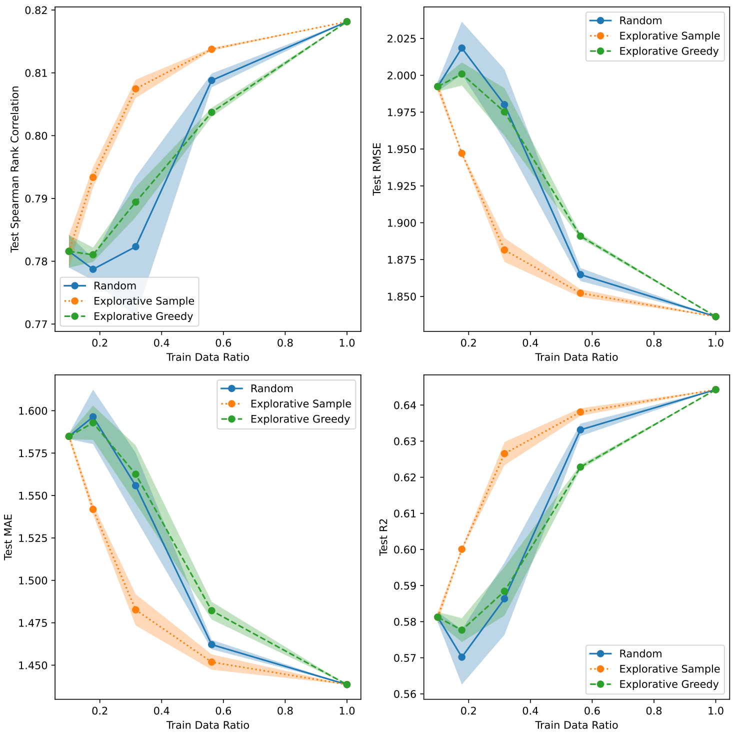

Figure N: Active learning results for AAV/Random using CNN Dropout uncertainty.

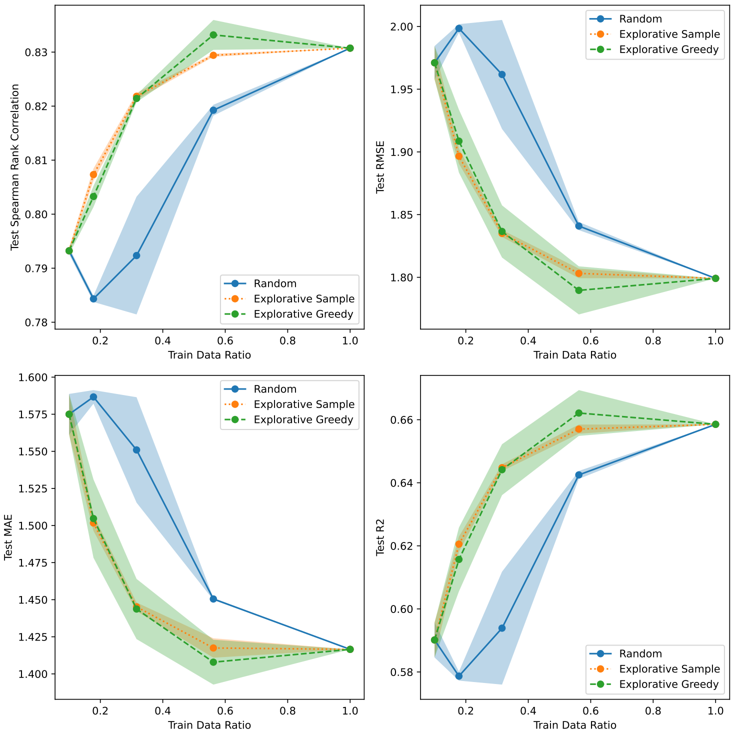

Figure O: Active learning results for AAV/Random using CNN Ensemble uncertainty.

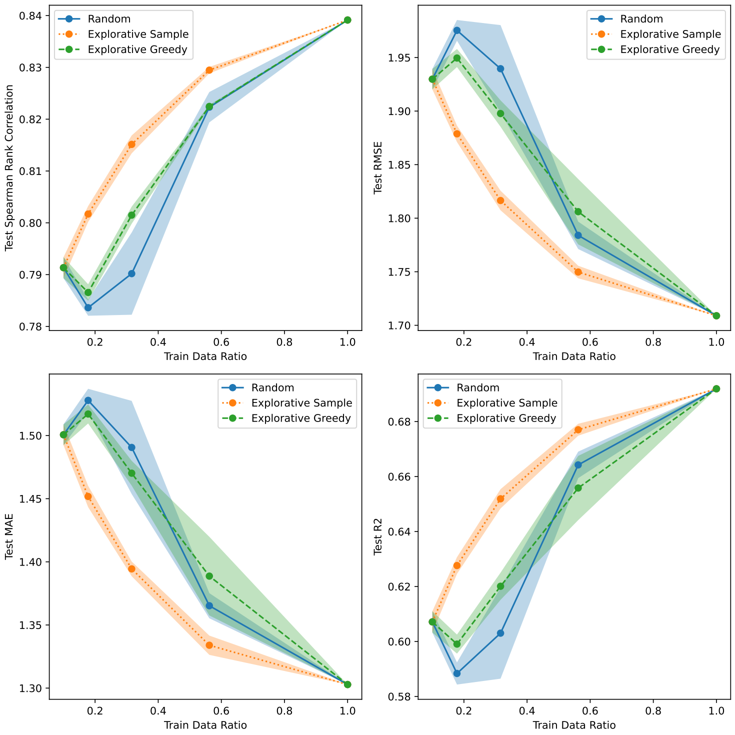

Figure P: Active learning results for AAV/Random using CNN Evidential uncertainty.

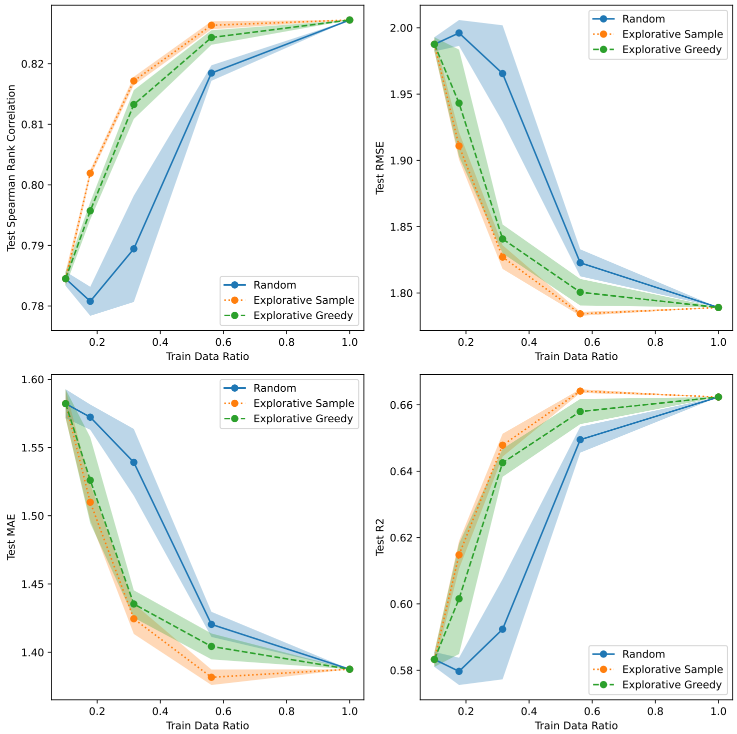

Figure Q: Active learning results for AAV/Random using CNN MVE uncertainty.

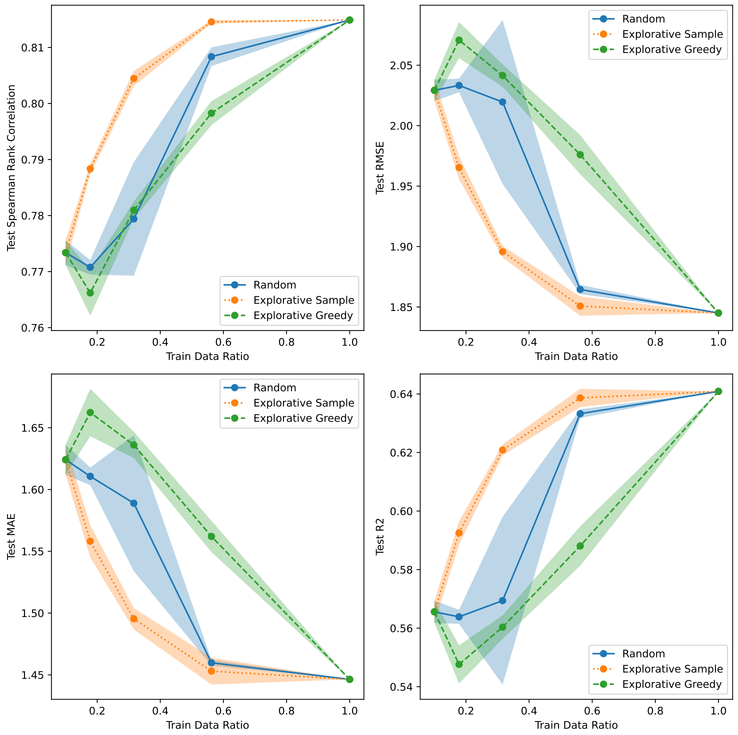

Figure R: Active learning results for AAV/Random using CNN SVI uncertainty.

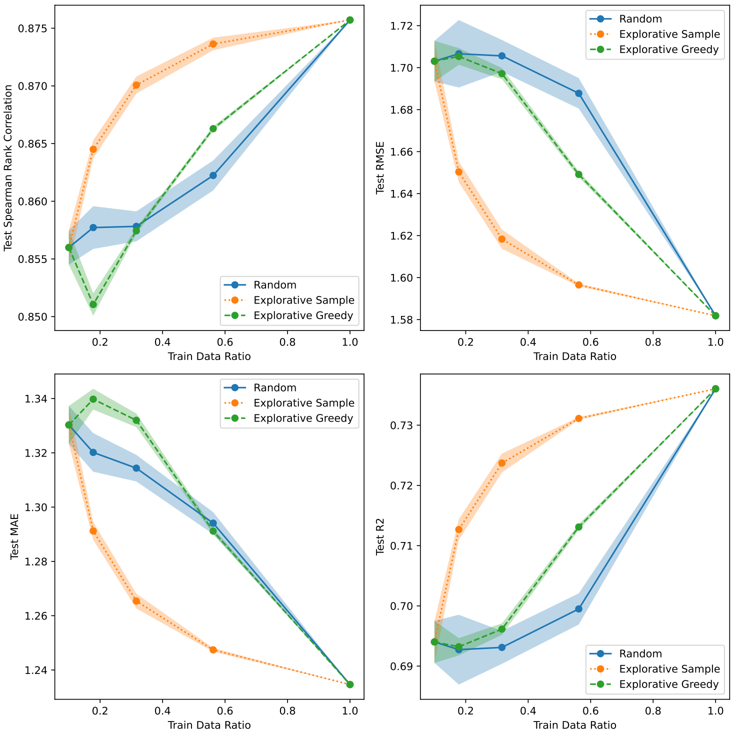

Figure S: Active learning results for AAV/Random using Linear Bayesian Ridge uncertainty.

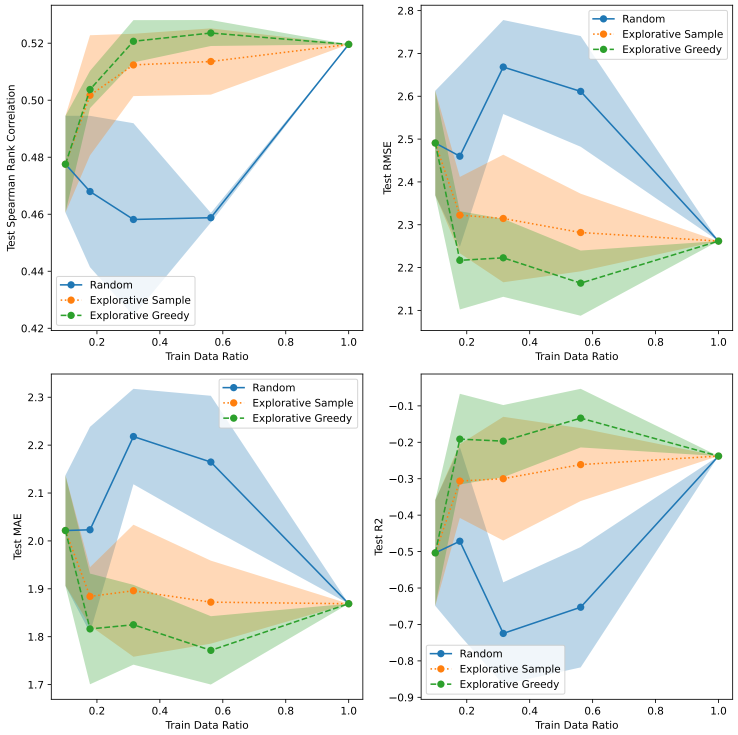

Figure T: Active learning results for AAV/7 vs. Rest using CNN Dropout uncertainty.

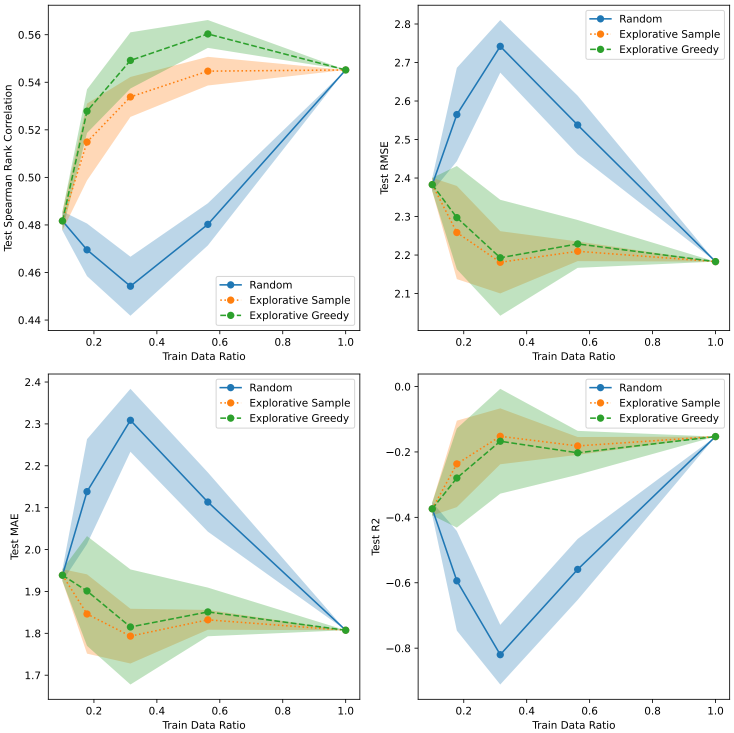

Figure U: Active learning results for AAV/7 vs. Rest using CNN Ensemble uncertainty.

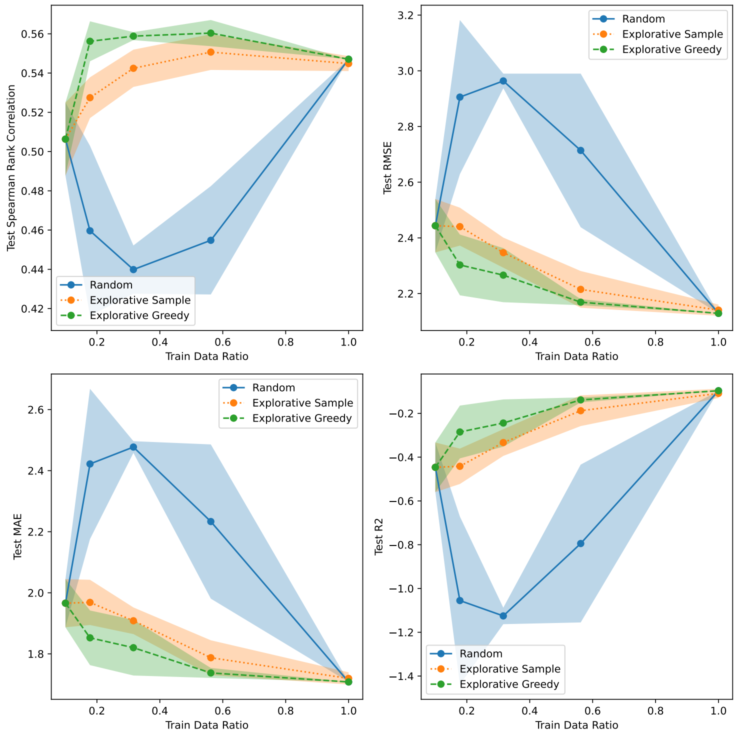

Figure V: Active learning results for AAV/7 vs. Rest using CNN Evidential uncertainty.

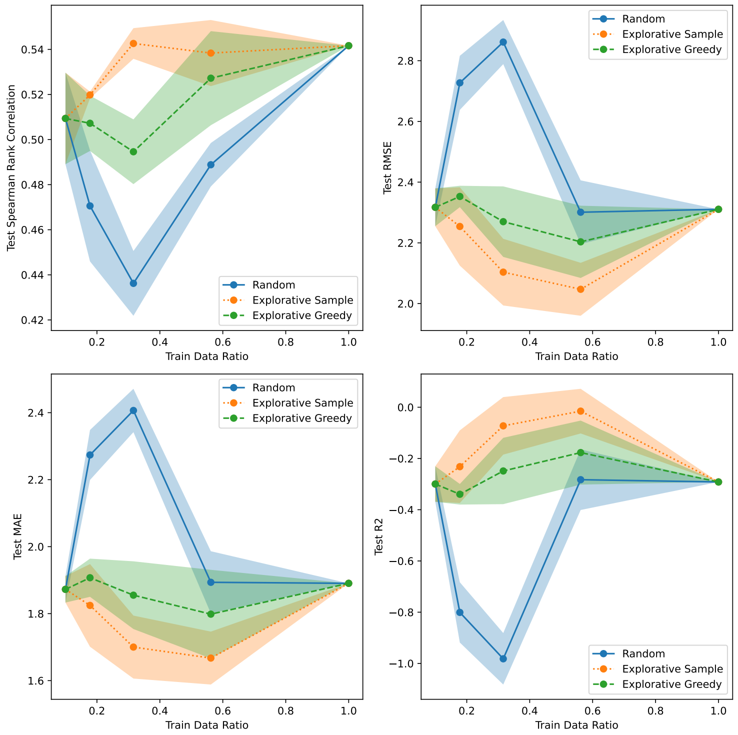

Figure W: Active learning results for AAV/7 vs. Rest using CNN MVE uncertainty.

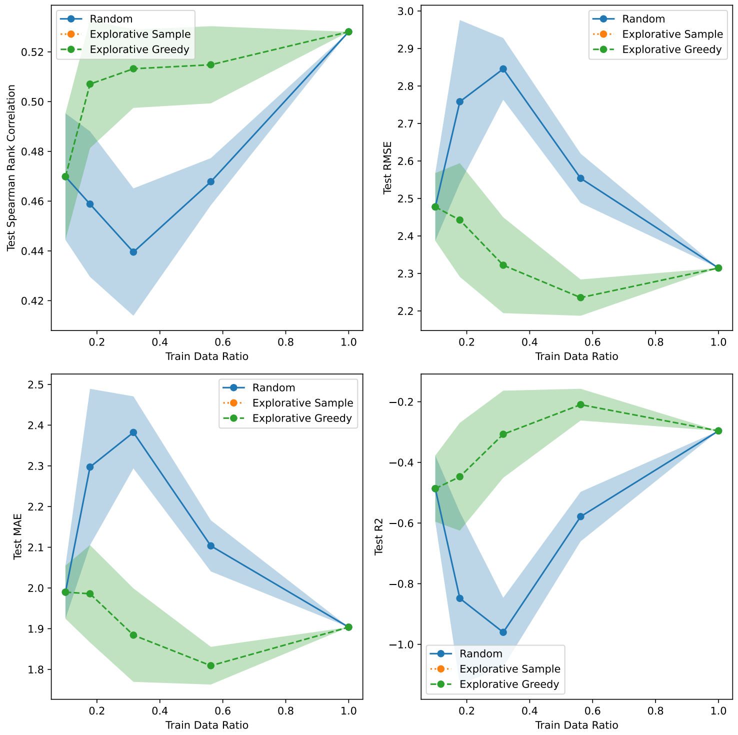

Figure X: Active learning results for AAV/7 vs. Rest using CNN SVI uncertainty.

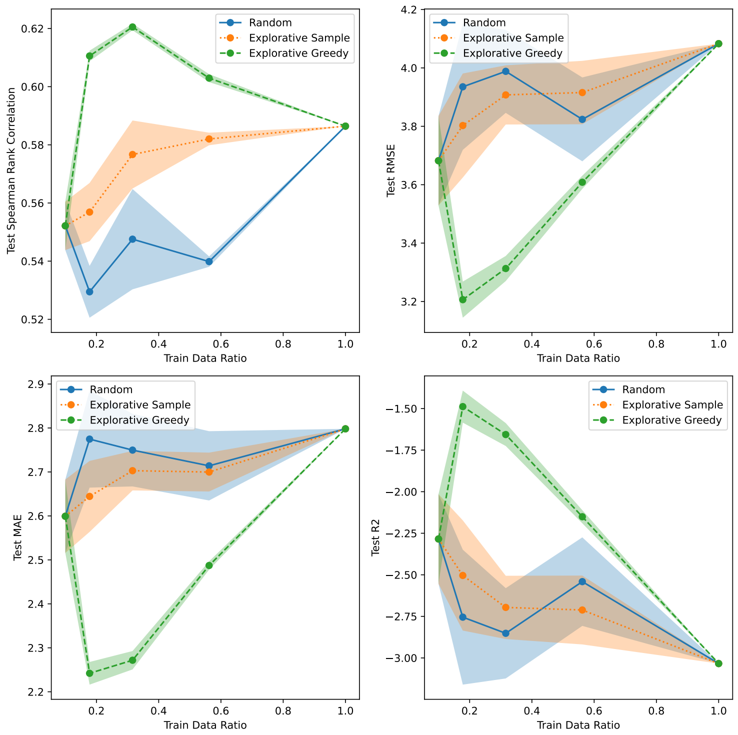

Figure Y: Active learning results for AAV/7 vs. Rest using Linear Bayesian Ridge uncertainty.

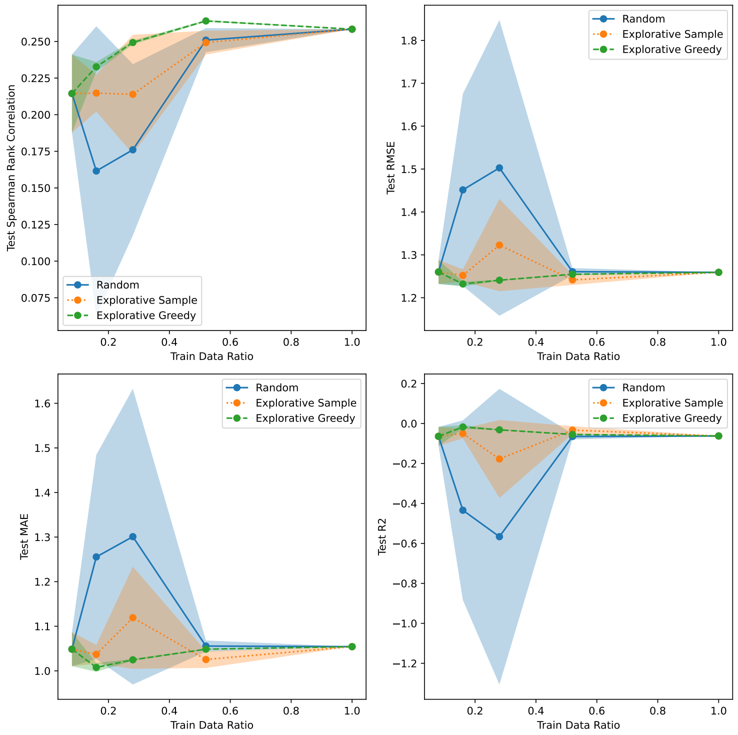

Figure Z: Active learning results for GB1/1 vs. Rest using CNN Dropout uncertainty.

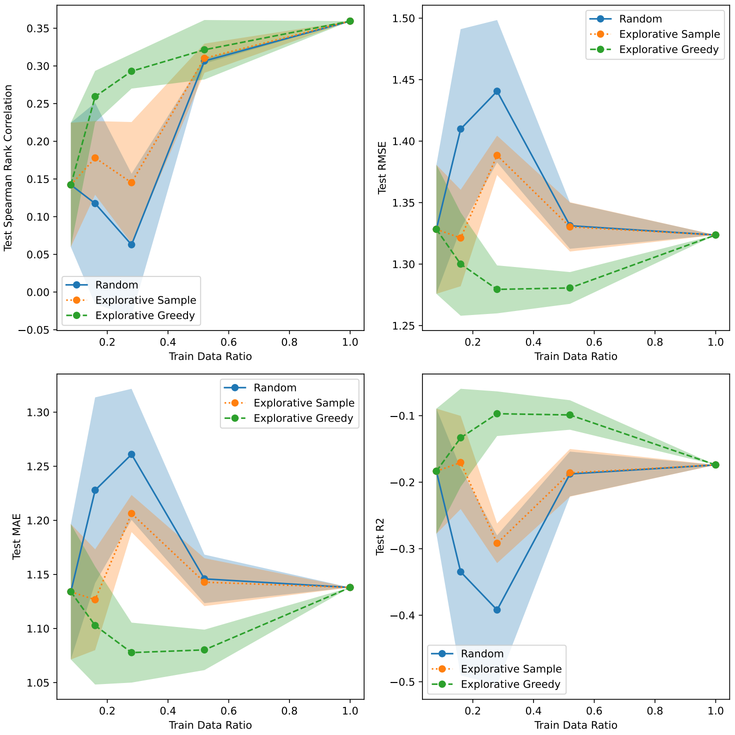

Figure AA: Active learning results for GB1/1 vs. Rest using CNN Ensemble uncertainty.

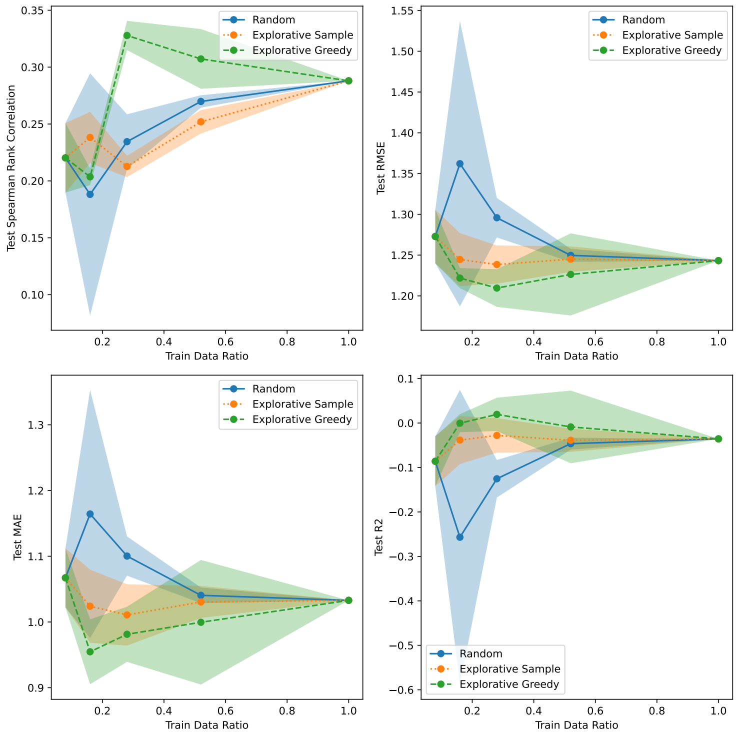

Figure AB: Active learning results for GB1/1 vs. Rest using CNN Evidential uncertainty.

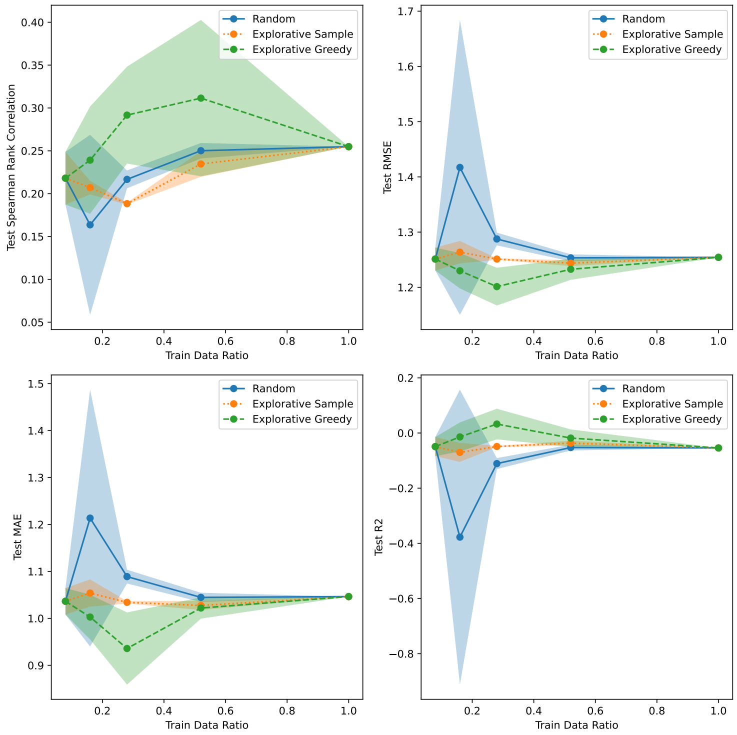

Figure AC: Active learning results for GB1/1 vs. Rest using CNN MVE uncertainty.

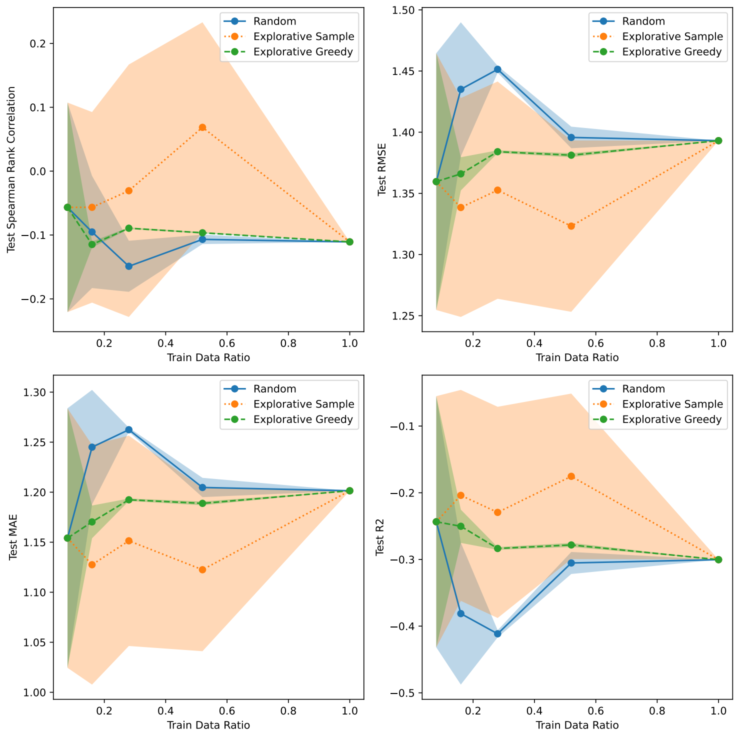

Figure AD: Active learning results for GB1/1 vs. Rest using CNN SVI uncertainty.

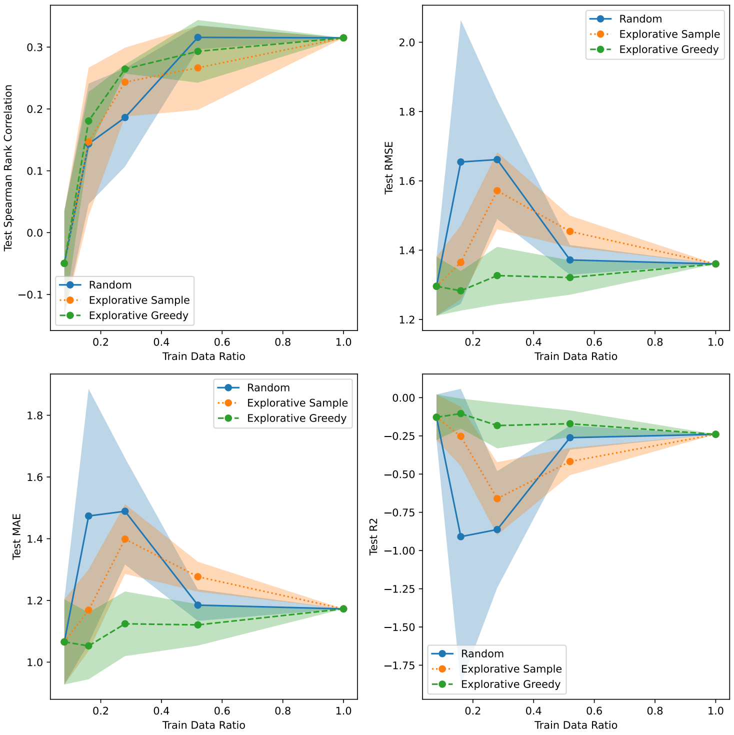

Figure AE: Active learning results for GB1/1 vs. Rest using GP Continuous uncertainty.

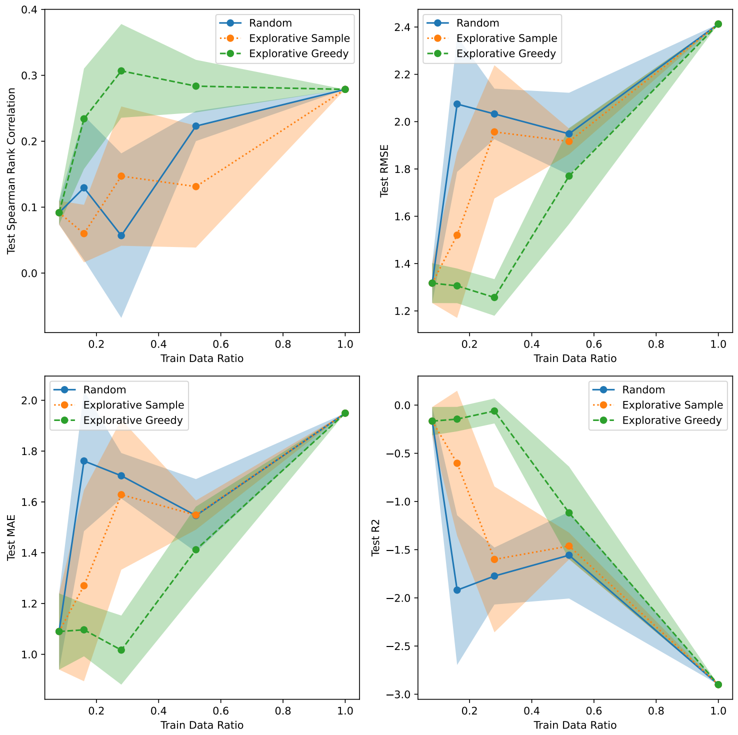

Figure AF: Active learning results for GB1/1 vs. Rest using Linear Bayesian Ridge uncertainty.

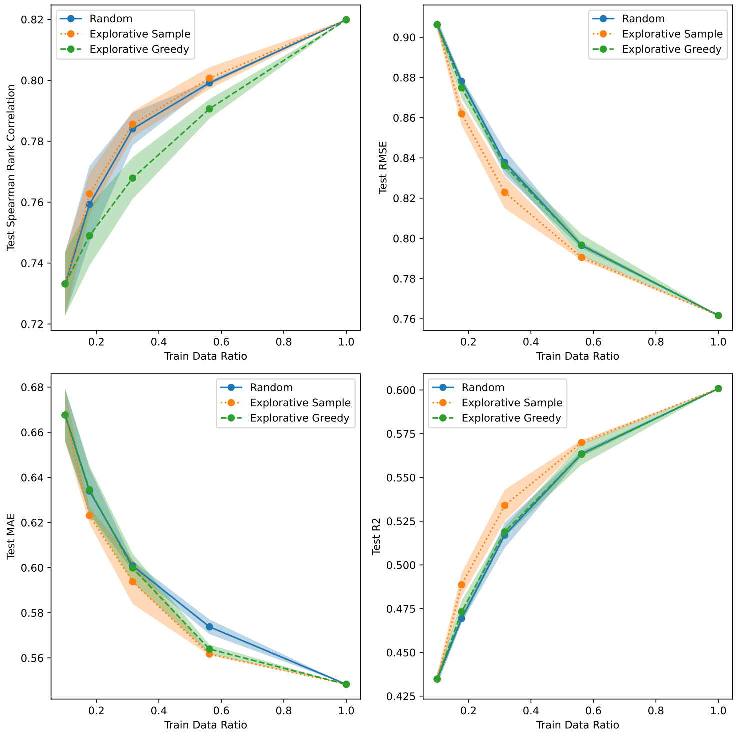

Figure AG: Active learning results for GB1/Random using CNN Dropout uncertainty.

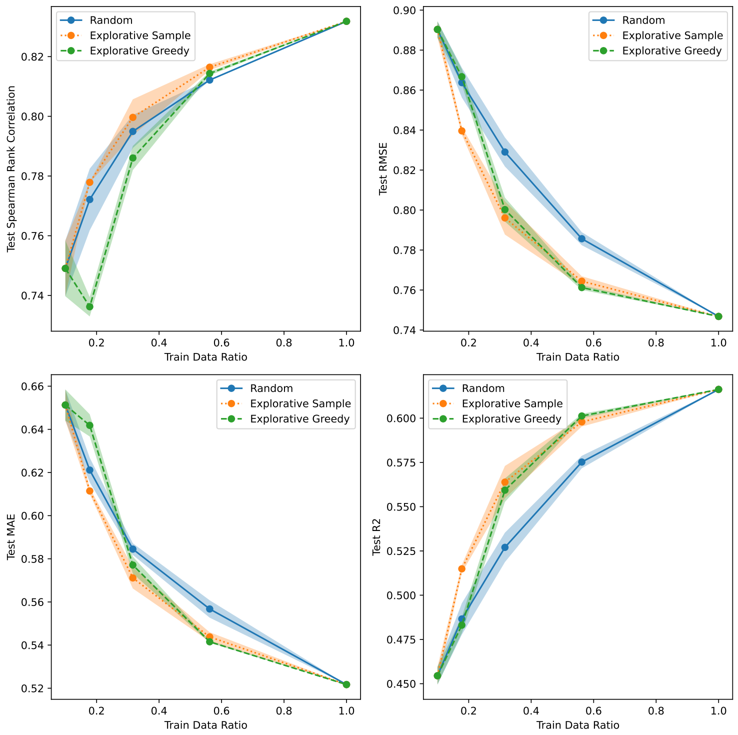

Figure AH: Active learning results for GB1/Random using CNN Ensemble uncertainty.

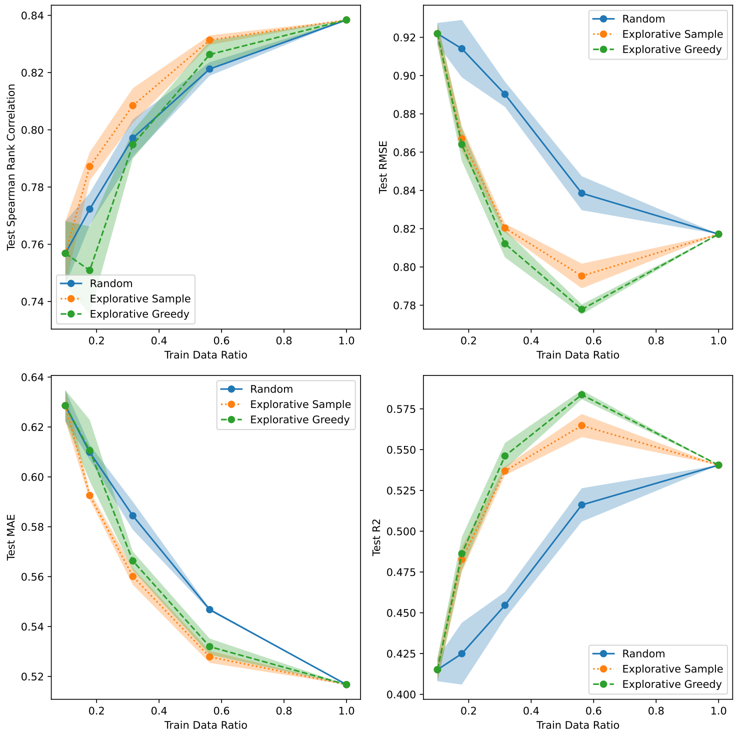

Figure AI: Active learning results for GB1/Random using CNN Evidential uncertainty.

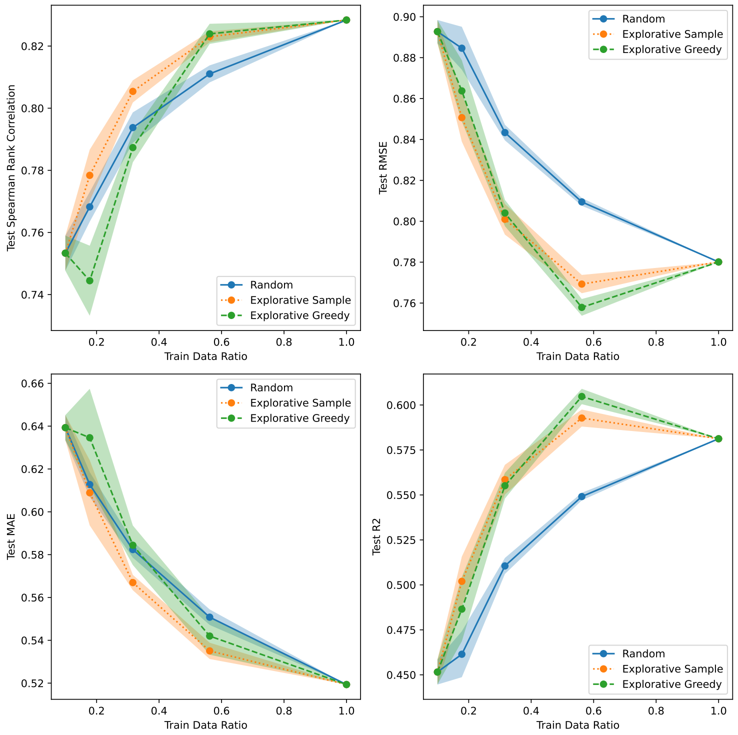

Figure AJ: Active learning results for GB1/Random using CNN MVE uncertainty.

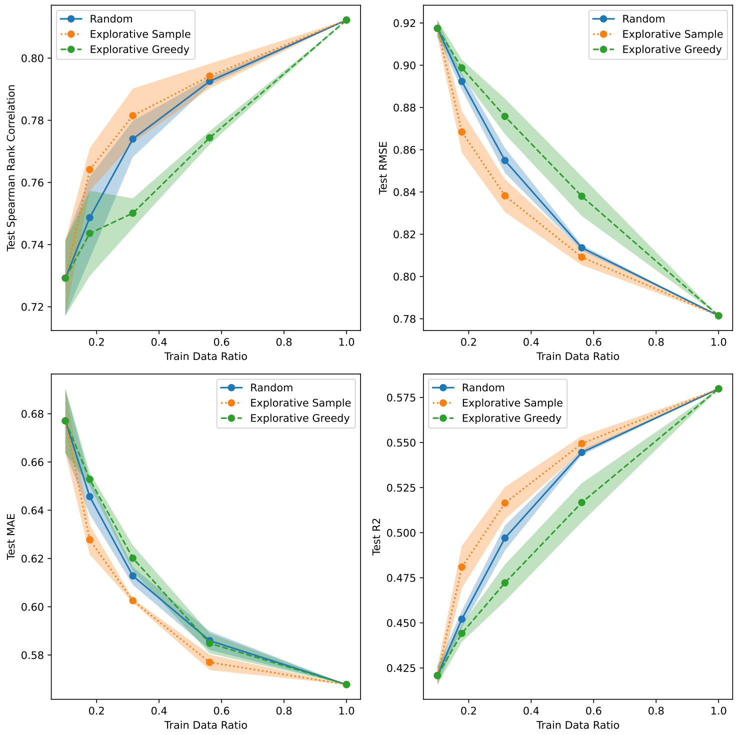

Figure AK: Active learning results for GB1/Random using CNN SVI uncertainty.

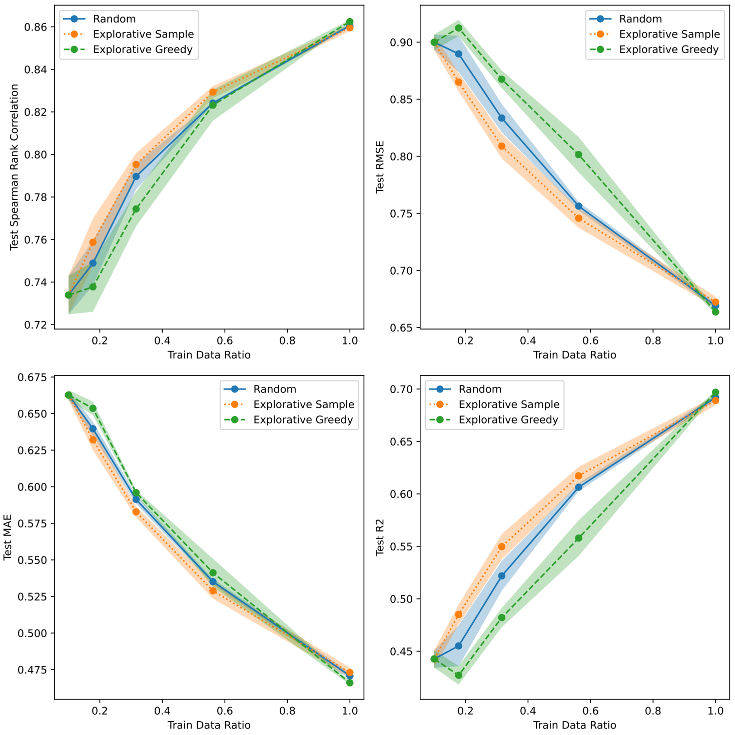

Figure AL: Active learning results for GB1/Random using GP Continuous uncertainty.

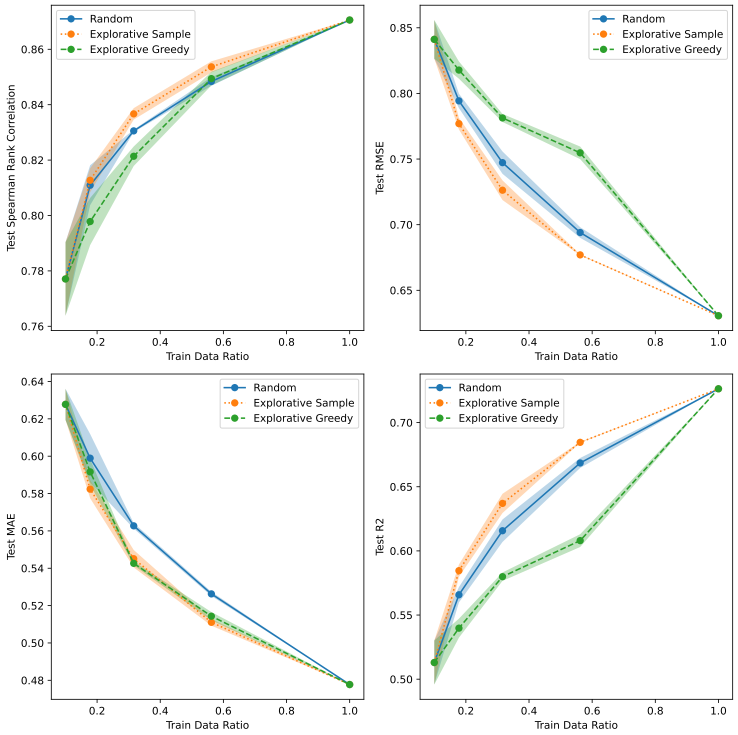

Figure AM: Active learning results for GB1/Random using Linear Bayesian Ridge uncertainty.

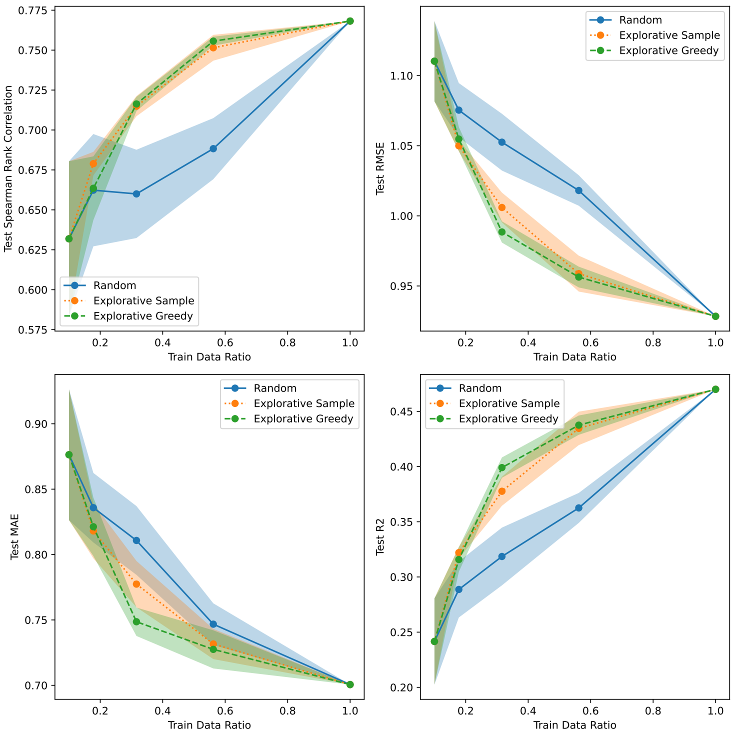

Figure AN: Active learning results for GB1/3 vs. Rest using CNN Dropout uncertainty.

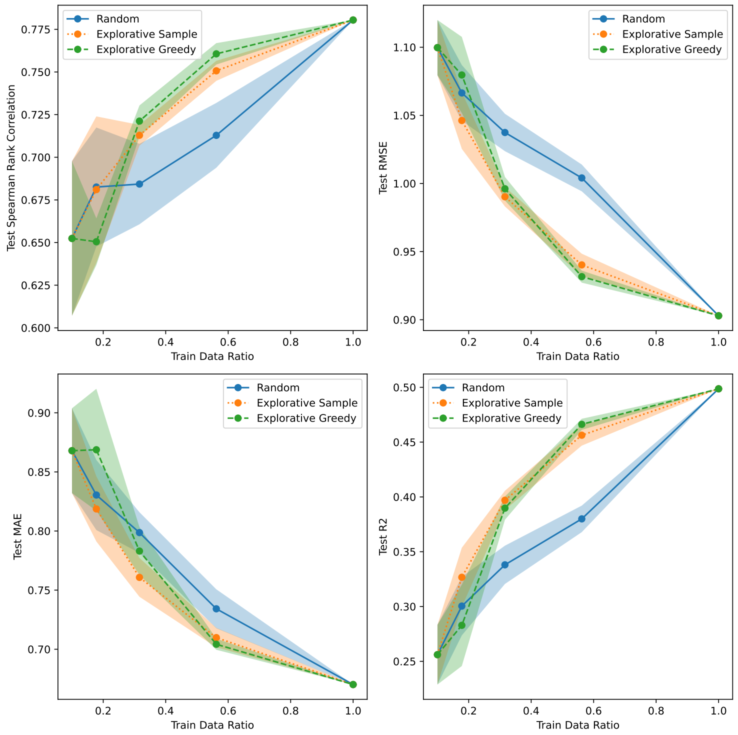

Figure AO: Active learning results for GB1/3 vs. Rest using CNN Ensemble uncertainty.

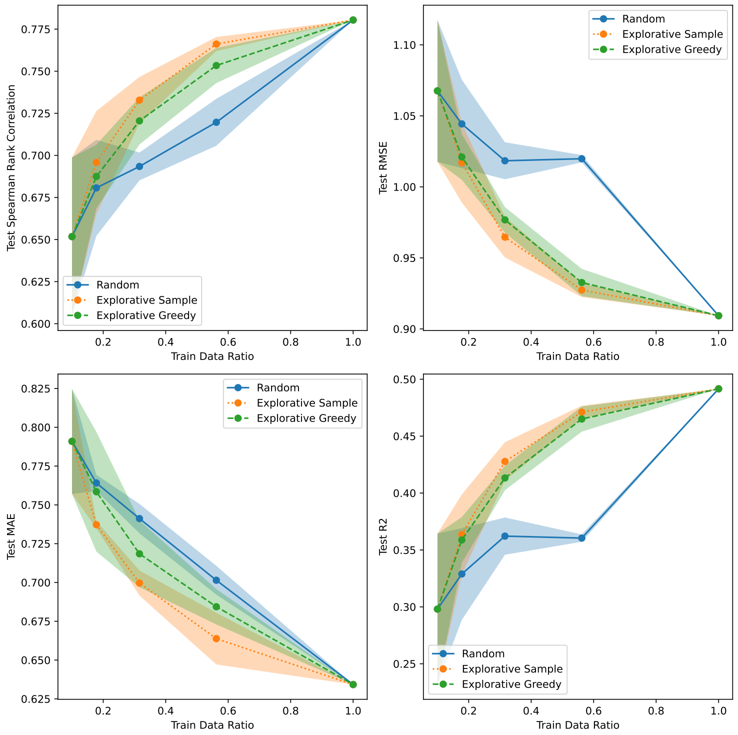

Figure AP: Active learning results for GB1/3 vs. Rest using CNN Evidential uncertainty.

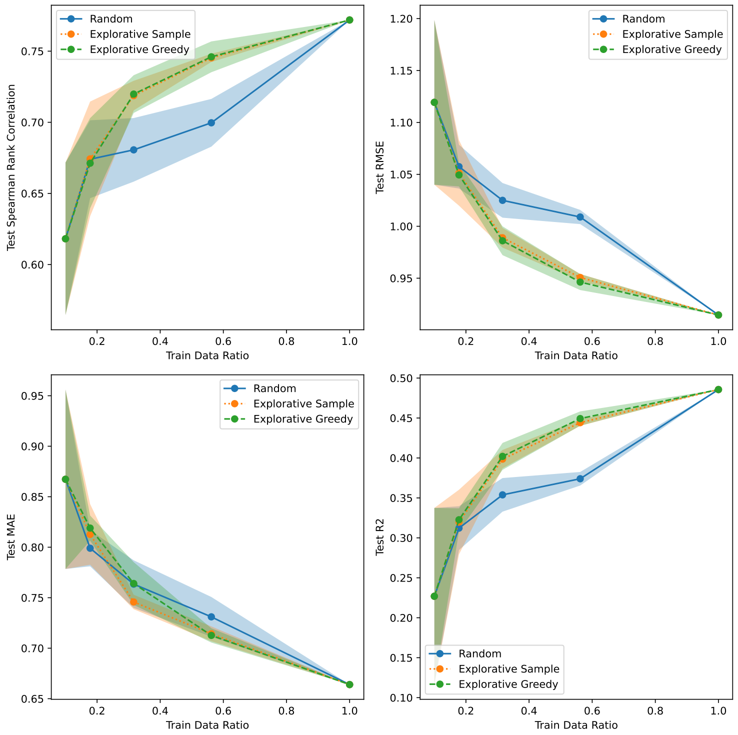

Figure AQ: Active learning results for GB1/3 vs. Rest using CNN MVE uncertainty.

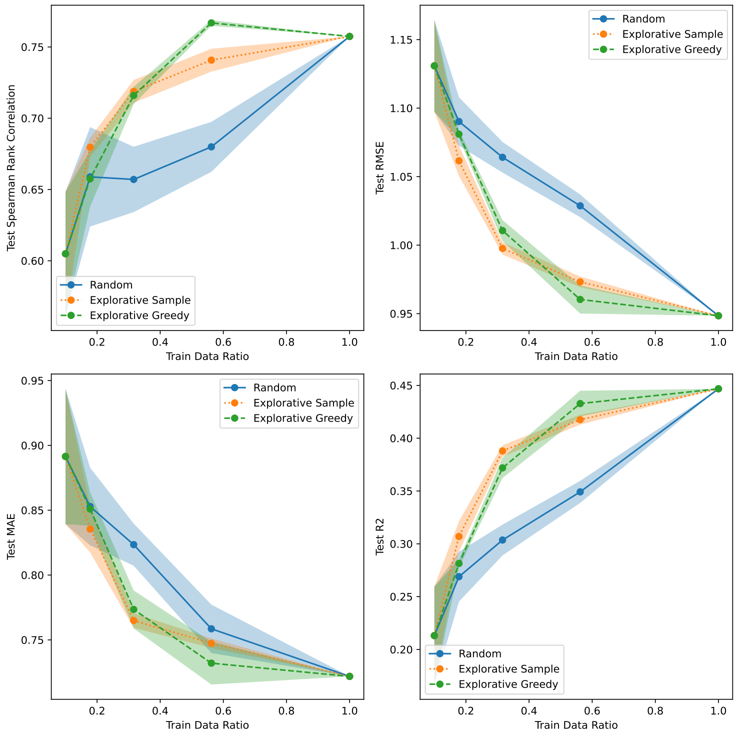

Figure AR: Active learning results for GB1/3 vs. Rest using CNN SVI uncertainty.

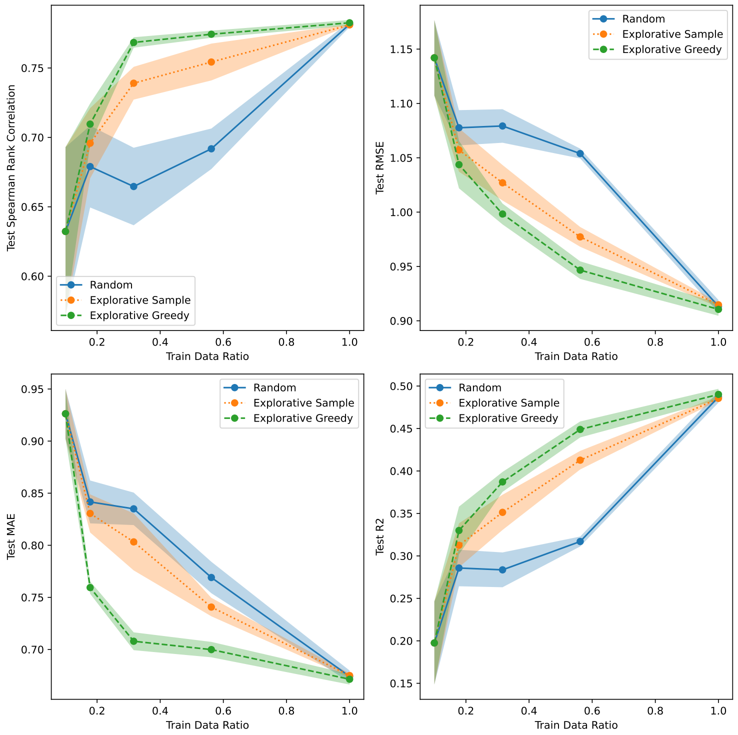

Figure AS: Active learning results for GB1/3 vs. Rest using GP Continuous uncertainty.

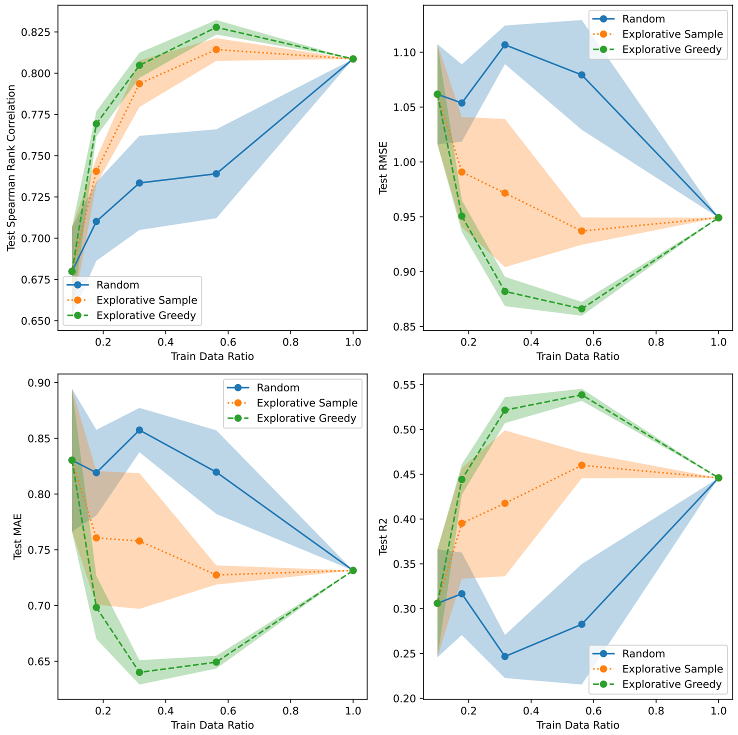

Figure AT: Active learning results for GB1/3 vs. Rest using Linear Bayesian Ridge uncertainty.

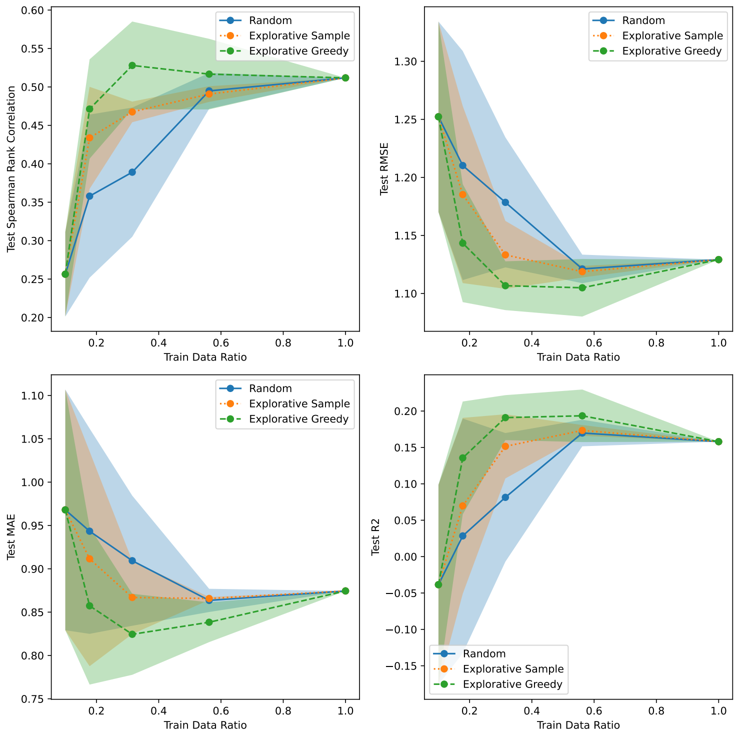

Figure AU: Active learning results for GB1/2 vs. Rest using CNN Dropout uncertainty.

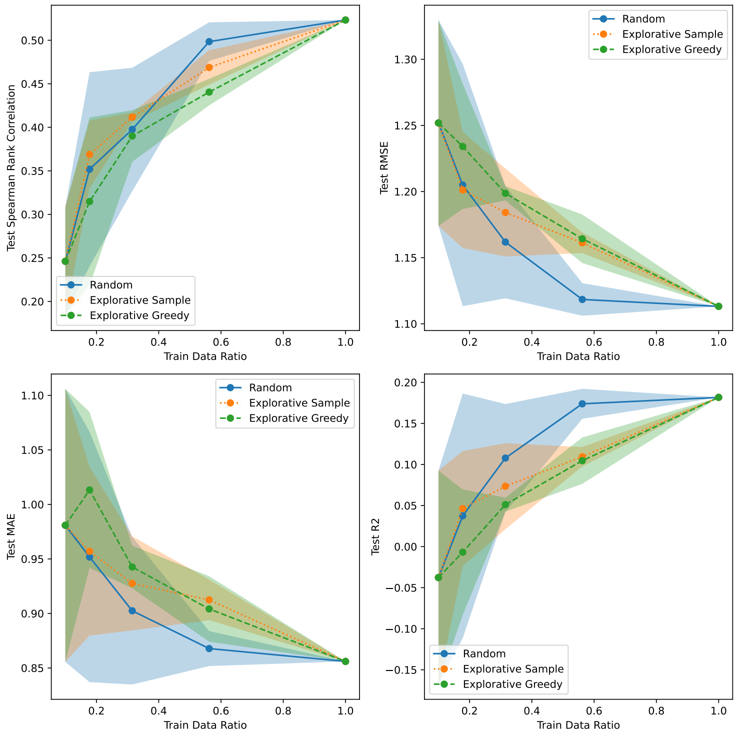

Figure AV: Active learning results for GB1/2 vs. Rest using CNN Ensemble uncertainty.

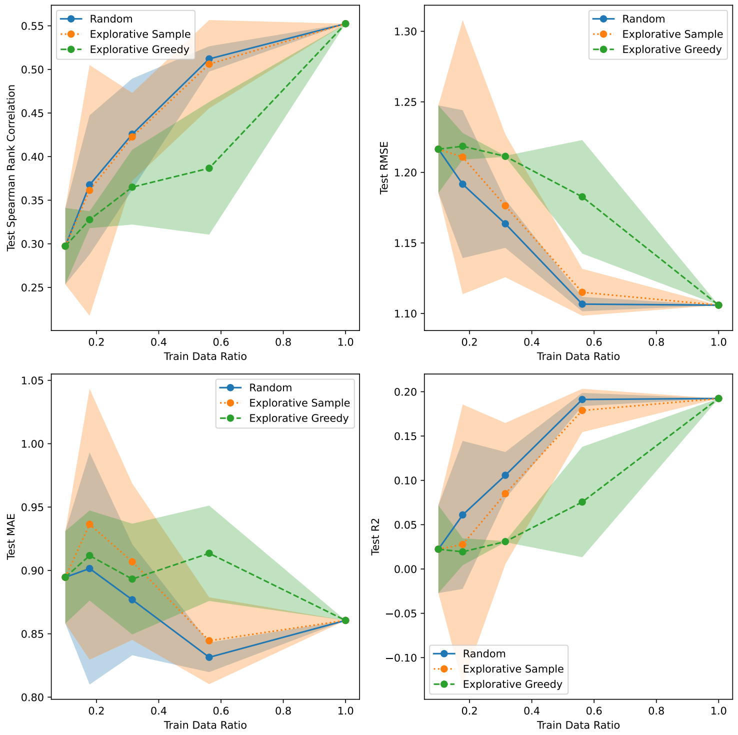

Figure AW: Active learning results for GB1/2 vs. Rest using CNN Evidential uncertainty.

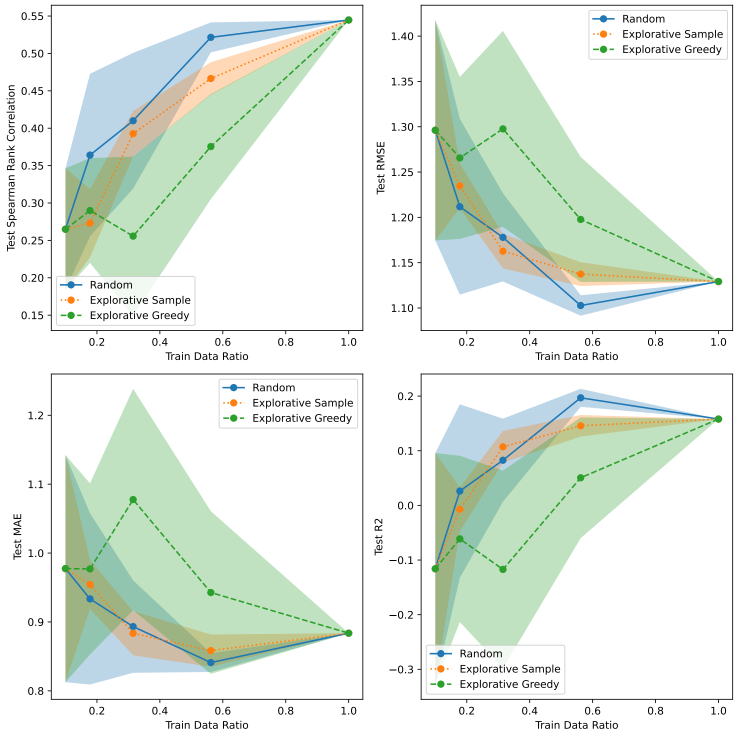

Figure AX: Active learning results for GB1/2 vs. Rest using CNN MVE uncertainty.

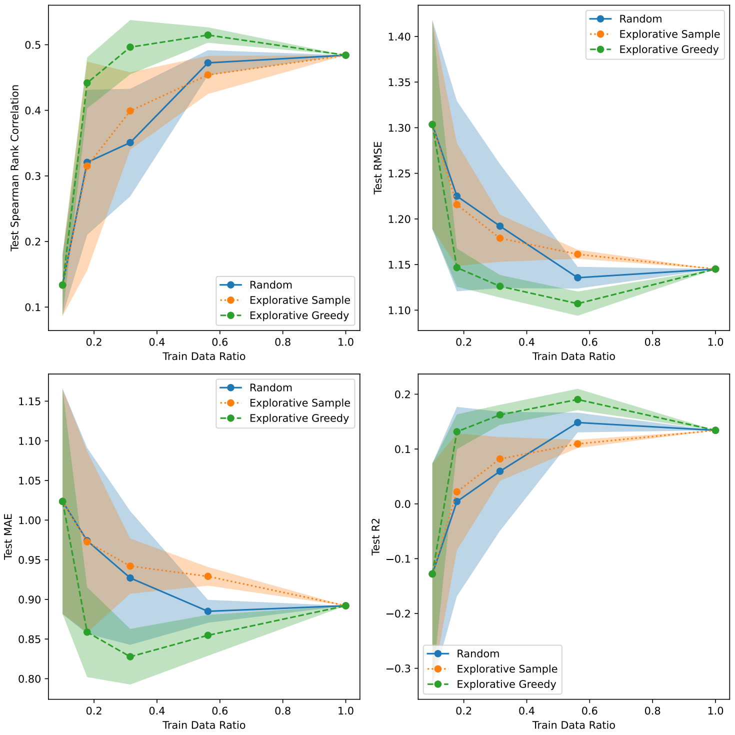

Figure AY: Active learning results for GB1/2 vs. Rest using CNN SVI uncertainty.

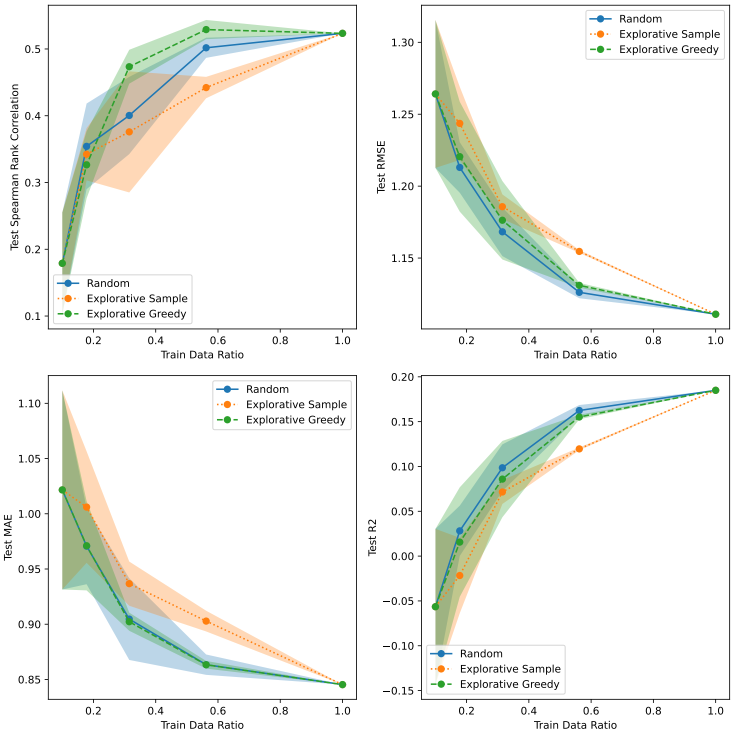

Figure AZ: Active learning results for GB1/2 vs. Rest using GP Continuous uncertainty.

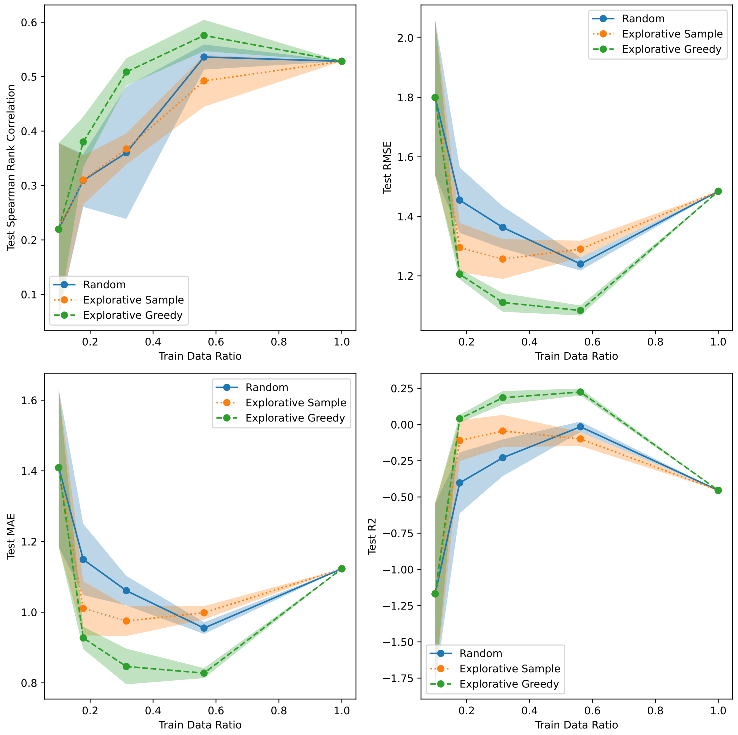

Figure BA: Active learning results for GB1/2 vs. Rest using Linear Bayesian Ridge uncertainty.

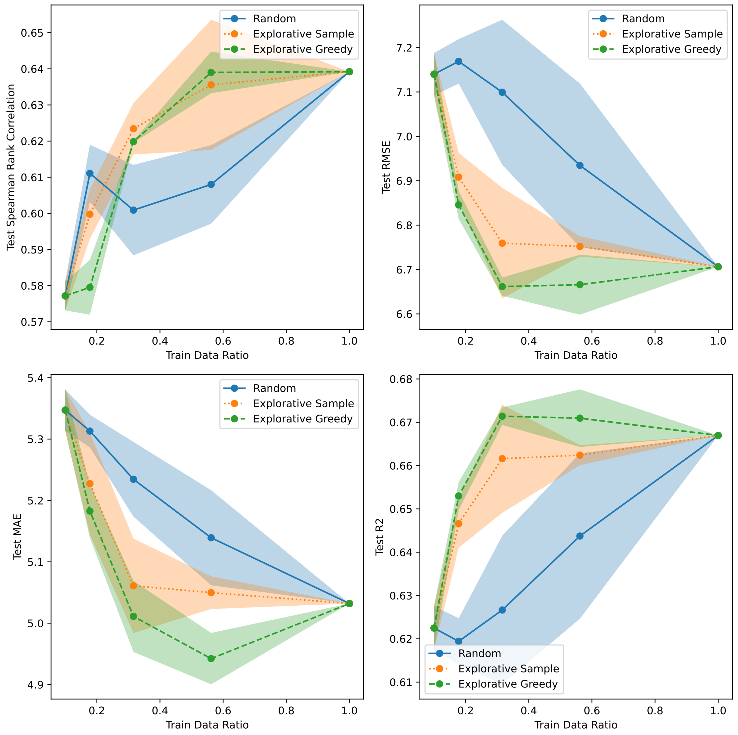

Figure BB: Active learning results for Meltome/Random using CNN Dropout uncertainty.

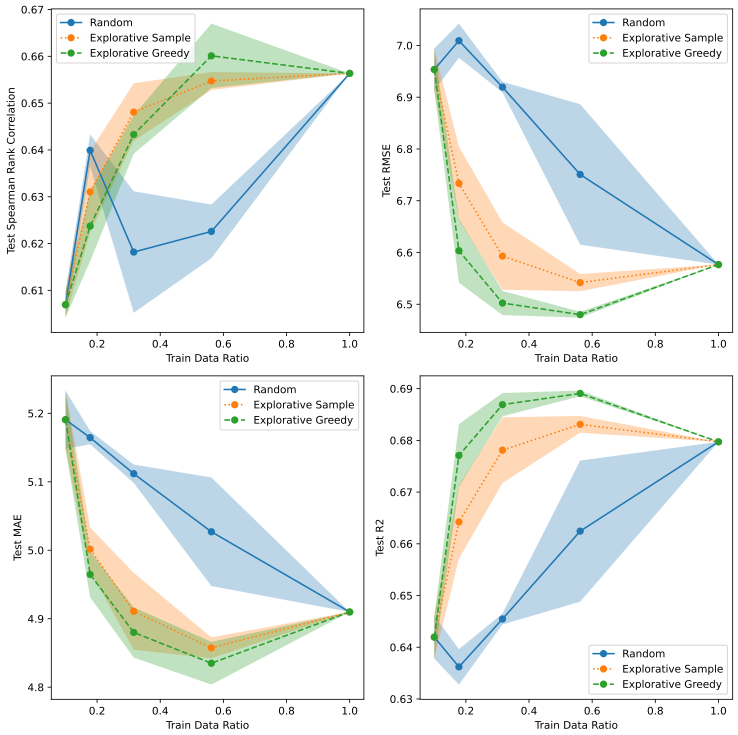

Figure BC: Active learning results for Meltome/Random using CNN Ensemble uncertainty.

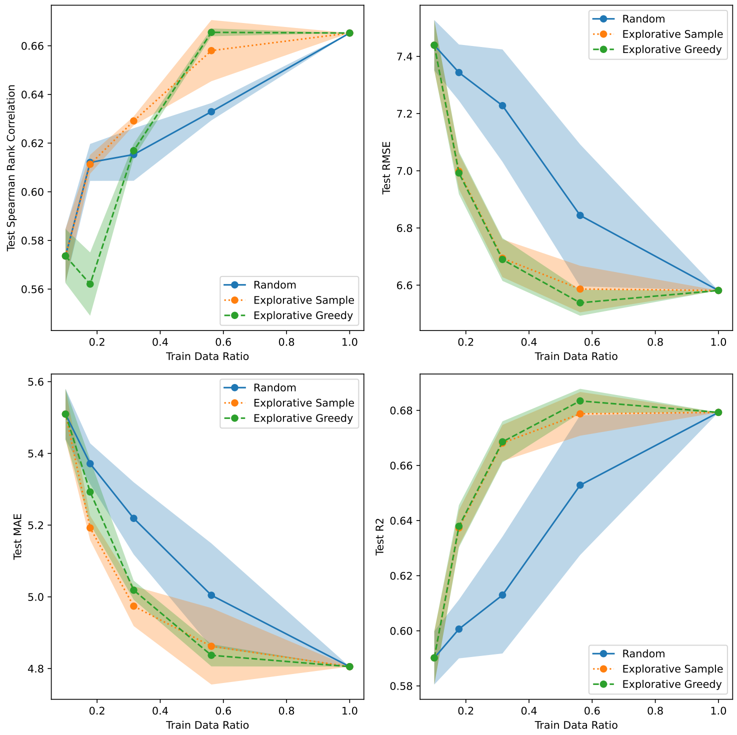

Figure BD: Active learning results for Meltome/Random using CNN Evidential uncertainty.

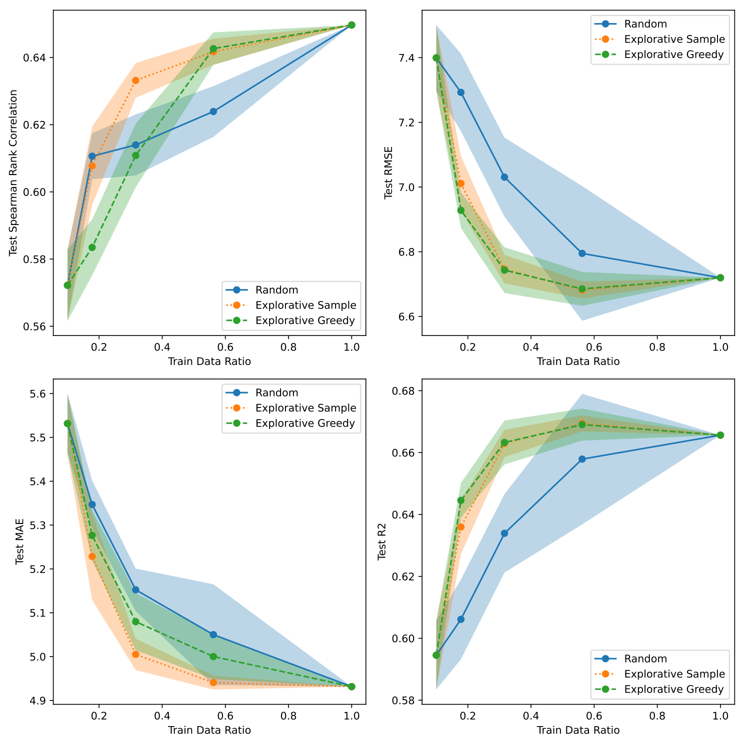

Figure BE: Active learning results for Meltome/Random using CNN MVE uncertainty.

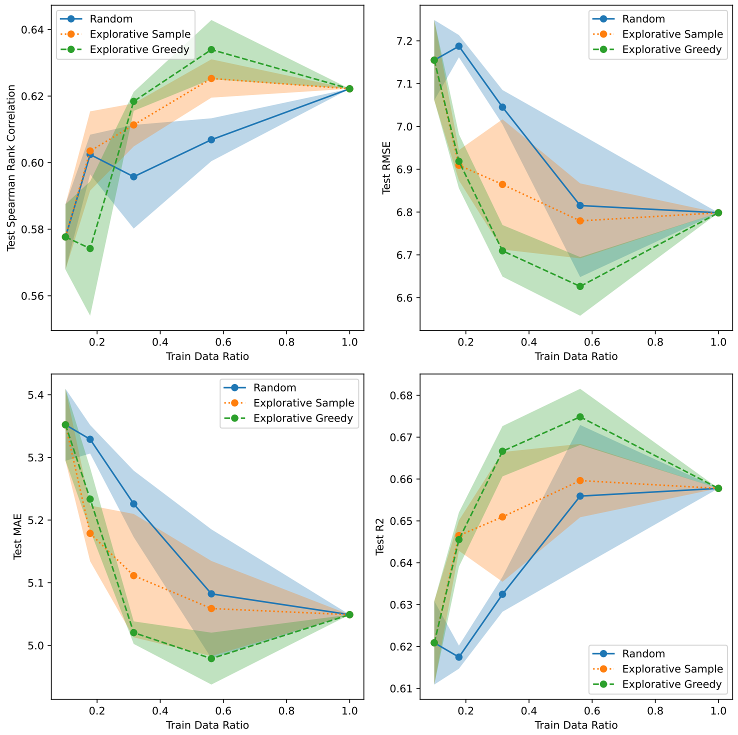

Figure BF: Active learning results for Meltome/Random using CNN SVI uncertainty.

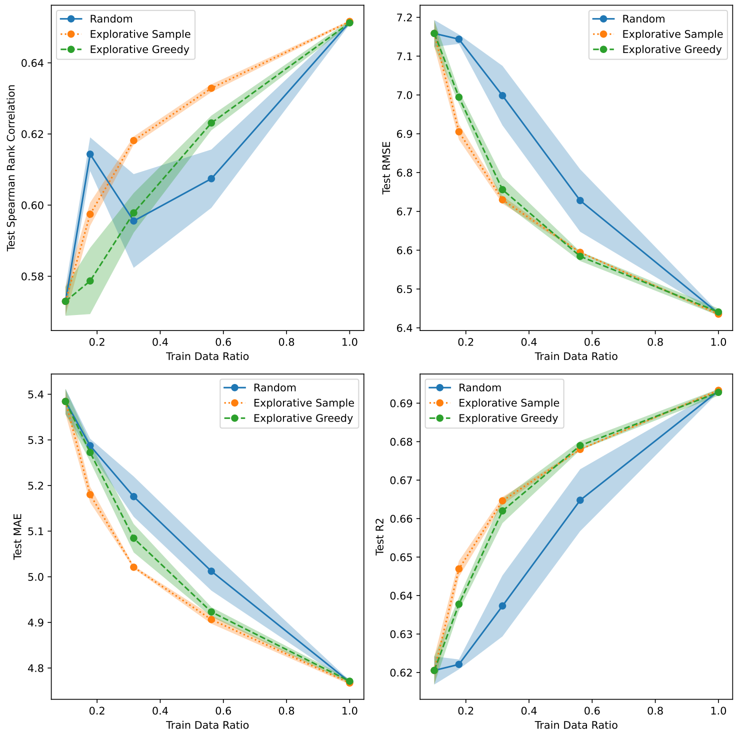

Figure BG: Active learning results for Meltome/Random using GP Continuous uncertainty.

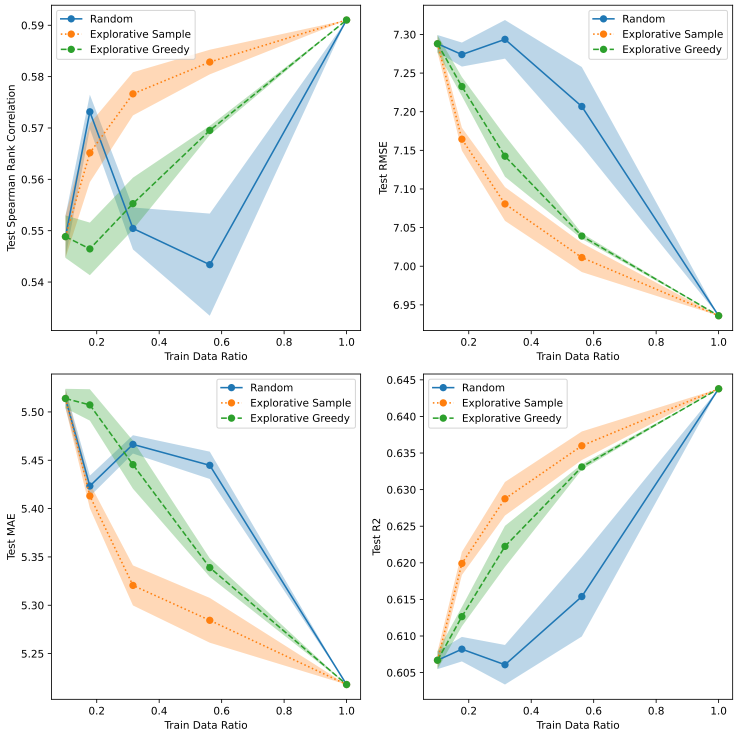

Figure BH: Active learning results for Meltome/Random using Linear Bayesian Ridge uncertainty.
