# Supplementary material for: Benchmarking uncertainty quantification for protein engineering
Source: PLoS Comput Biol. 2025 Jan 7;21(1):e1012639. doi: 10.1371/journal.pcbi.1012639 (PMC11741572; doi:10.1371/journal.pcbi.1012639)
Supplement: Attachment — Submitted filename: protein_uq_plos_compbio-reviewer_responses.pdf [file pcbi.1012639.s002.pdf]

Dear Yang,

Thank you very much for submitting your manuscript "Benchmarking uncertainty quantification for protein engineering" for consideration at PLOS Computational Biology.

As with all papers reviewed by the journal, your manuscript was reviewed by members of the editorial board and by several independent reviewers. In light of the reviews (below this email), we would like to invite the resubmission of a significantly-revised version that takes into account the reviewers' comments.

We cannot make any decision about publication until we have seen the revised manuscript and your response to the reviewers' comments. Your revised manuscript is also likely to be sent to reviewers for further evaluation.

When you are ready to resubmit, please upload the following:

[1] A letter containing a detailed list of your responses to the review comments and a description of the changes you have made in the manuscript. Please note while forming your response, if your article is accepted, you may have the opportunity to make the peer review history publicly available. The record will include editor decision letters (with reviews) and your responses to reviewer comments. If eligible, we will contact you to opt in or out.

[2] Two versions of the revised manuscript: one with either highlights or tracked changes denoting where the text has been changed; the other a clean version (uploaded as the manuscript file).

Important additional instructions are given below your reviewer comments.

Please prepare and submit your revised manuscript within 60 days. If you anticipate any delay, please let us know the expected resubmission date by replying to this email. Please note that revised manuscripts received after the 60-day due date may require evaluation and peer review similar to newly submitted manuscripts.

Thank you again for your submission. We hope that our editorial process has been constructive so far, we apologize for the long time it took, and we welcome your feedback at any time. Please don't hesitate to contact us if you have any questions or comments.

Sincerely,

Rachel Kolodny
Academic Editor
PLOS Computational Biology

Nir Ben-Tal
Section Editor

PLOS Computational Biology

**********************

Reviewer's Responses to Questions

**Comments to the Authors:**
**Please note here if the review is uploaded as an attachment.**

**Reviewer #1:**

This paper is about quantifying the ability of different protein sequence-based models to accurately predict uncertainty in different protein design data regimes and modalities. The authors tried difference sequence representations, model architectures and uncertainty quantification methods across FLIP benchmark tasks. Although the amount of experiments involved in this work is impressive, the paper is unfortunately written in a way that mostly lists those dense results, hence making it difficult to get any insight or grasp the relationship between them. Moreover, some of the paper's claims could benefit from better statistical analysis support.

**Major comments**

The paper is results-dense and would benefit from some curation of metrics and/or models that would make it easier to ingest. For example, I suspect that RMSE and correlation are highly correlated as well as calibration plot and uncertainty correlation, but they both have their own distinct figures. Some of these results could be moved to supplementary.

While it may be true that RMSE and Spearman rho are correlated with one another, this is not a given. Similarly for calibration and uncertainty correlation, we think it is important to show a variety of metrics to property assess prediction and uncertainty performance. Each metric is designed to assess something different about the model, and we want to encourage readers to consider many metrics when making choices about how to model uncertainty. To the related point in your minor questions below, we have added additional discussion about some of these metrics to the main text to provide more context about why each one is important.

The figures are hard to read or interpret since they tend to have too much information and don't use the space efficiently by repeating the same axis and legend that end up using all the space. For example, the bar plots in Figure 4 are hard to read, especially the B panel for models that have close to zero correlation that seems to be missing.

This is a good point. We have revised Figure 4 to use a single shared x-axis and legend, which allows us to make the figure slightly larger and easier to read. Note that some of the bars that appear to be missing are actually not present at all, due to computational constraints. This is mentioned in the caption and explained in more detail in Section 4.6. We considered combining

axes for Figures 2 and 3, but given that the x-axis ranges are not consistent, combining them into a single x-axis would result in some of the plots getting squished into a narrower range, making them harder to read. We also considered using shared legends for these plots, but the shapes in each subfigure require separate legends because the names of the split types for each landscape are different (e.g. 3 vs. rest for GB1 and 7 vs. rest for AAV are represented by the same shape).

Can the authors comment on the apparent strong relationship found between the coverage and width metrics for the same task across different models? It almost points toward models not better quantifying uncertainty per point, but just being more confident in general. It almost feels that a simple re-scaling of the uncertainties on a calibration set from training sets would make those trends and claims vanish.

It's true that the trend of increasing coverage with increasing width could point to a general under/over confidence for the different types of models. We've added a note about this to the main text. It's also a good point that these results would be affected by post-hoc calibration. Given that there are many ways to do post-hoc calibration and that our benchmark already involved so many experiments, we decided that was outside the scope of this work, but we've mentioned that in our discussion and conclusions sections to encourage others to consider this in future work.

Claims about models being better than others on task are hard to support without any statistical testing. The fact that using different starting seeds for training the CNN model gives similar performance across Figure 4 almost indicates that some of the trends found in previous figures could also vanish. Also, the active learning experiments with the random acquisition function seem to indicate that a simple re-sampling of the training data could be used to better estimate confidence intervals on metrics and support claims about model performance versus each other. Can the authors re-sample their training data to generate different training data and get a better estimation of their metrics? Can they also choose at least 3-4 different starting points in BO and active learning to not let the starting 10% dictate the conclusion?

We agree, and for this reason we have avoided any use of the word "significant" when making comparisons between methods. The points shown in Figures 2 and 3, like the bars in Figure 4, are the average of multiple runs with different starting seeds, as mentioned in the respective captions. We chose to omit error bars on these points in Figures 2 and 3 because it made the plots more crowded and difficult to read.

The error regions in Figures 5 and 6 for AL and BO are already derived from different random seeds for different sub-samplings of the dataset, and different sub-sampling is not possible for the earlier figures since we train on the whole dataset at once to generate those metrics.

This is a good point that the results might be affected by our choice to start at 10% of the training data. We've repeated the AL experiments starting with 1% and 5% of the training data and added these results to the SI (Figures S6 and S7), as well as added a comment about this to the main text. We found that the different starting points resulted in similar trends to what we

observed at 10%.

Can the authors comment on the expected relationship between the different metrics? It is not clear why the miscalibration curve should be plotted against the RMSE.

There is not necessarily an expected relationship between the metrics since they all attempt to measure different things. In some sense, it is arbitrary which metrics we choose to plot against one another; it is just a way of visualizing the results in two dimensions. We plotted the miscalibration area vs. the RMSE (Figure 2) to compare model uncertainty calibration to model prediction accuracy. In Figure 3, we plotted coverage vs. width to compare two uncertainty metrics to one another.

**Minor comments**

The authors do cite their uncertainty metrics, but given how central they are to the paper, can they describe them more?

Yes, we have added more description of the uncertainty metrics to Section 4.7, along with descriptions of settings where each metric might be more important than others.

I might have missed these details, but what are the data used for active learning and BO. Is it the designed AAV or just the random? Would it make more sense to start from sampled and then sample from designed? (or maybe it is the case)

Our AL and BO experiments use only the random splits for each of the three landscapes. We realize now that this was only mentioned in the captions of Figures 5 and 6, so we have added a note about this to Sections 4.8 and 4.9 of the Methods. Starting from the random dataset and sampling from designed could indeed be an interesting experiment. In the active learning case, one would need to think carefully about how to choose an appropriate test set when doing sampling in this way (e.g. holding out a subset of the designed sequences for a test set and sampling from the other designed sequences to add to the training set). The splits in the FLIP benchmark dataset are not set up to facilitate such an experiment, but this could be an interesting subject of future work. We have added a sentence to the conclusion mentioning this.

In the active learning experiments, are the models getting more confident as they get more details?

Sometimes yes, but in other cases the mean uncertainty on the test set actually increases, which could correspond to the model adjusting for being overconfident at lower training data sizes. We have added a figure to the SI (Figure S5) some some description in the main text to illustrate this.

A summary table of the different rankings of selected models across tasks would be helpful since the paper has so many results that it is hard to keep track of performance across different regimes and modalities. Ideally, these rankings would be derived from robust statistical testing.

We considered several options for how to summarize the results (e.g. ranking by area under the correlation vs. data size curve for active learning). However, we think that a summary table with rankings may falsely imply to readers a simpler conclusion than what we actually observed. If a reader of this paper were to take away that a particular uncertainty method is better than others because it was ranked higher in a summary table, that would be missing a core theme of the paper, which is that any comparison between methods is heavily dependent on which metrics are used, the dataset, and the data splitting / degree of domain shift.

Some curves are not visible in Figure 6 since they are hidden by others.

If one zooms in close enough on the figure, the overlap is not exact, but we've added a note to the caption of Figure 6 to point out that the greedy and UCB strategies have nearly identical and overlapping performance for a few of the experiments to avoid confusion.

---

**Reviewer #2:**
This paper considers the problem of uncertainty prediction for the problem of protein engineering. The paper reports extensive experimentation, with the goal to evaluate different types of predictors, and specifically evaluate their uncertainty predictions. The experiment is conducted in three different types of tasks, with varying degree of domain shift between the training data and test data. The uncertainty prediction is evaluated using different metrics, and through two downstream applications, namely, active learning and Bayesian optimization. The results of the experiments show that:
1. There is no single method that performs better across all tasks.
2. Current uncertainty predictors are not always useful for downstream tasks like active learning (where they are useful in some cases), and Bayesian optimization (where a greedy, uncertainty-agnostic approach performs better).
3. Representing proteins using the ESM language model embeddings rather than one-hot encoding, improves results in some cases but not all.

The main conclusion is that uncertainty predictors cannot be assumed to work out of the box for protein engineering tasks and need to be further developed, and/or carefully evaluated per task.

Strengths:

1. The paper performs an extensive and thorough evaluation of the methods under different varying conditions, and gives both a detailed description of each setup and the big picture of the status of current methods.

2. The conclusion is a useful and practical contribution to the community that often considers relying on such predictions of uncertainty.

Weaknesses:

1. The paper does not present a novel model or evaluation methodology, and is a relatively straightforward implementation of various experiments. In my view this should not prevent the paper from being accepted as the experimentation is thorough and therefore valuable to the community.

2. The results do not show a clear winning method that can directly inform practitioners on ways to improve their research on downstream tasks. However, like I mentioned above, there is value in empirically demonstrating the limitations of current models, which should serve as a warning for practitioners of downstream tasks, and an invitation to researchers to perform more research on uncertainty prediction.

3. There are a few issues that were not clear to me. See questions below. I believe that fixing those issues, would make the paper publishable.

Questions:

1. The representation and CNN architecture is not clear to me. What dimension exactly is being averaged in the ESM embeddings? At the end, what is the dimension of the protein representation both for ESM and one-hot encoding? Is it constant or varying with sequence length? Why do you need a CNN to process this representation, as opposed to a fully connected MLP? Is there some local invariance or smoothness property that should be captured by convolutions? If so over what dimension?

The output of the ESM-1b model is a tensor of size L x 1280, where L is the number of amino acids in the sequence. We average over the amino acids to obtain the fixed-length representation vector of size 1280. We've added a sentence to Section 4.3 to clarify this. This is one of the methods used in the FLIP benchmark paper and is a standard output option implemented for the ESM models (using the `--include mean` argument in the esm-extract script provided by Facebook/Meta Research.

The dimension of the protein representation for OHE (after convolution, a linear layer, and max pooling) is 2048. The dimension of the representation at the same point in the model for ESM data is 2560. These are both constant regardless of input sequence length.

The CNN is designed to capture local structures from the OHE representations in the sequence dimension. This is a good point that the CNN architecture may not be necessary for the ESM representation since averaging over amino acids has already given us a global representation, so replacing the convolutional layer with a linear layer might perform better for ESM. However, we have chosen to keep the architecture consistent to compare the effect of changing the representation. Even with a suboptimal architecture, the ESM approach outperforms the OHE approach in about half of all cases.

2. It is not clear from the description in section 4.6 that all methods use the same number of samples in evaluation. If this is the case it should be stated, and if not, it should be discussed and justified.

We used n = 10 samples for both dropout and SVI, and an ensemble size of n = 5. We chose a smaller number for the ensemble approach because the cost scales linearly with the size of the ensemble. In other words, sampling 10 times at inference time does not add much cost to the model, compared to the overhead of training 5 separate members of an ensemble before making 5 separate predictions at inference time. These points are stated in Section 4.6.

3. Some methods were not fully described, for example it was mentioned that the evidential CNN uses a loss termed L^R, without elaborating.

Thank you for pointing this out. We added a citation to the paper that defines this loss term.

3. Section 4.7 is confusing in listing the different metrics. First it describes MAE and R^2 metrics which I failed to see in any of the epxeriment results. Second, it states there are four uncertainty

metrics but I could only count three (ro_unc, coverage, AUCE). Third, the description of the coverage states that the range is 4\sigma/R rather than 4\sigma, however in figure 3 these are shown on different axes (coverage vs. width/R) - I'm not sure what's going on there.

Please see Section 4 of the SI for the results using the MAE and R^2 metrics (we also refer to Tables S1-S22 in the main text). Regarding the uncertainty metrics, it appears there may be some confusion about the difference between the coverage metric and the width metric. While somewhat related, these are distinct metrics. We've made some modifications to the text of this section to try to clarify that.

Some general remarks:

1. The fact that the linear model and GP are performing better than the CNN suggest that maybe there is not enough training data for a deep learning method to work. Are there larger datasets that can be tested?

While the linear models and GPs do perform well (in some cases even better than the CNNs), many of the active learning results for CNNs in Figure 5 display plateauing behavior as we increase the dataset size from 10% up to 100% of the training data (~66k samples in the case of the AAV landscape). This plateauing behavior demonstrates the diminishing returns of adding additional training data, which suggests that the CNNs have sufficient data to learn.

2. I'm not sure there is a way to do this better than the thorough experiments setup already presented, but in the evaluation through downstream tasks (active learning and Bayesian optimization), there is still some conflation between the quality of the prediction and the quality of the uncertainty predictions. It might be beneficial to try to compare a predictor where the uncertainty is computed exactly from ground truth data. This can serve as some kind of upper bound on the performance.

This is a good point that there is conflation between the quality of the predictions and the quality of the uncertainty predictions. Unfortunately, this is unavoidable. There is no "ground truth" data available for uncertainties. Even if experimental uncertainties from the lab were known and homogenous/uniform, this would only account for the aleatoric uncertainty (data-dependent or irreducible uncertainty, i.e. noise) in the data. This would not account for the epistemic uncertainty (model-dependent uncertainty, i.e. bias and variance), which is often the more relevant type of uncertainty for active learning since this uncertainty can be reduced by gathering more data. One could calculate a "ground truth uncertainty" by using the actual prediction errors in place of the uncertainties, but this would not be a very meaningful baseline since true values are not known at inference time in practice. As you state, this could give an upper bound on performance, but we do not feel that this would contribute to our analysis since our main concern is whether uncertainty-based methods can outperform random or greedy baselines.

---

**Have the authors made all data and (if applicable) computational code underlying the findings in their manuscript fully available?**
The PLOS Data policy requires authors to make all data and code underlying the findings described in their manuscript fully available without restriction, with rare exception (please refer to the Data Availability Statement in the manuscript PDF file). The data and code should be provided as part of the manuscript or its supporting information, or deposited to a public repository. For example, in addition to summary statistics, the data points behind means, medians and variance measures should be available. If there are restrictions on publicly sharing data or code —e.g. participant privacy or use of data from a third party—those must be specified.

Reviewer #1: Yes

Reviewer #2: Yes

---

PLOS authors have the option to publish the peer review history of their article (what does this mean?). If published, this will include your full peer review and any attached files.

If you choose “no”, your identity will remain anonymous but your review may still be made public.

**Do you want your identity to be public for this peer review?** For information about this choice, including consent withdrawal, please see our Privacy Policy.

Reviewer #1: No

Reviewer #2: No

Figure Files:

While revising your submission, please upload your figure files to the Preflight Analysis and Conversion Engine (PACE) digital diagnostic tool, https://pacev2.apexcovantage.com. PACE helps ensure that figures meet PLOS requirements. To use PACE, you must first register as a user. Then, login and navigate to the UPLOAD tab, where you will find detailed instructions on how to use the tool. If you encounter any issues or have any questions when using PACE, please email us at figures@plos.org.

Data Requirements:

Please note that, as a condition of publication, PLOS' data policy requires that you make available all data used to draw the conclusions outlined in your manuscript. Data must be deposited in an appropriate repository, included within the body of the manuscript, or uploaded

as supporting information. This includes all numerical values that were used to generate graphs, histograms etc.. For an example in PLOS Biology see here: http://www.plosbiology.org/article/info%3Adoi%2F10.1371%2Fjournal.pbio.1001908#s5.

Reproducibility:

To enhance the reproducibility of your results, we recommend that you deposit your laboratory protocols in protocols.io, where a protocol can be assigned its own identifier (DOI) such that it can be cited independently in the future. Additionally, PLOS ONE offers an option to publish peer-reviewed clinical study protocols. Read more information on sharing protocols at https://plos.org/protocols?utm_medium=editorial-email&utm_source=authorletters&utm_campaign=protocols
