# Supplementary material for: Benchmarking uncertainty quantification for protein engineering
Source: PLoS Comput Biol. 2025 Jan 7;21(1):e1012639. doi: 10.1371/journal.pcbi.1012639 (PMC11741572; doi:10.1371/journal.pcbi.1012639)
Supplement: Attachment — Submitted filename: protein_uq_plos_compbio-reviewer_responses2.pdf [file pcbi.1012639.s003.pdf]

Dear Yang,

Thank you very much for submitting your manuscript "Benchmarking uncertainty quantification for protein engineering" for consideration at PLOS Computational Biology. As with all papers reviewed by the journal, your manuscript was reviewed by members of the editorial board and by several independent reviewers. The reviewers appreciated the attention to an important topic. Based on the reviews, we would like to accept this manuscript for publication, but please modify the small changes the reviewer requested.

Please prepare and submit your revised manuscript within 30 days. If you anticipate any delay, please let us know the expected resubmission date by replying to this email.

When you are ready to resubmit, please upload the following:

[1] A letter containing a detailed list of your responses to all review comments, and a description of the changes you have made in the manuscript. Please note while forming your response, if your article is accepted, you may have the opportunity to make the peer review history publicly available. The record will include editor decision letters (with reviews) and your responses to reviewer comments. If eligible, we will contact you to opt in or out

[2] Two versions of the revised manuscript: one with either highlights or tracked changes denoting where the text has been changed; the other a clean version (uploaded as the manuscript file).

Important additional instructions are given below your reviewer comments.

Thank you again for your submission to our journal. We hope that our editorial process has been constructive so far, and we welcome your feedback at any time. Please don't hesitate to contact us if you have any questions or comments.

Sincerely,

Rachel Kolodny

Academic Editor

PLOS Computational Biology

Nir Ben-Tal

Section Editor

PLOS Computational Biology

****

A link appears below if there are any accompanying review attachments. If you believe any reviews to be missing, please contact ploscompbiol@plos.org immediately:

Reviewer's Responses to Questions

Comments to the Authors:
Please note here if the review is uploaded as an attachment.

Reviewer #1: The author has addressed most of my comments.

Regarding the statistical evidence discussion, I do understand that creating new splits is its own endeavor, and this paper is not about FLIP but about uncertainty quantification. However, since the author already did the hard work of training 5 models per split with different seeds, can they also report the standard deviation for all metrics in the supplementary table. This could help the reader have some context on how different models perform relative to each other in a given context

This is a good point. We have updated the manuscript to include all of these standard deviations. Specifically, each table S1-S22 in the Supporting Information that contained the mean values of the prediction/uncertainty metrics now has a corresponding table with the standard deviations of each metric. We now have renumbered the tables S1-S44, such that the odd-numbered tables are the old S1-S22 and contain the means, while the even-numbered tables are new and contain the standard deviations. Table S2 contains the standard deviations for S1, S4 contains the standard deviations for S3, etc. We also added a short paragraph to this section of the SI to explain where the standard deviations come from. Additionally, we added sentences to the “CNN Model Training Procedures” methods section to clarify our use of different initialization seeds, and we updated the reference in the results section to SI tables S1-S22 to reflect the additional tables for standard deviations.

Have the authors made all data and (if applicable) computational code underlying the findings in their manuscript fully available?
The PLOS Data policy requires authors to make all data and code underlying the findings

described in their manuscript fully available without restriction, with rare exception (please refer to the Data Availability Statement in the manuscript PDF file). The data and code should be provided as part of the manuscript or its supporting information, or deposited to a public repository. For example, in addition to summary statistics, the data points behind means, medians and variance measures should be available. If there are restrictions on publicly sharing data or code —e.g. participant privacy or use of data from a third party—those must be specified.

Reviewer #1: Yes

PLOS authors have the option to publish the peer review history of their article (what does this mean?). If published, this will include your full peer review and any attached files.

If you choose “no”, your identity will remain anonymous but your review may still be made public.

Do you want your identity to be public for this peer review? For information about this choice, including consent withdrawal, please see our Privacy Policy.

Reviewer #1: No

Figure Files:

While revising your submission, please upload your figure files to the Preflight Analysis and Conversion Engine (PACE) digital diagnostic tool, https://pacev2.apexcovantage.com. PACE helps ensure that figures meet PLOS requirements. To use PACE, you must first register as a user. Then, login and navigate to the UPLOAD tab, where you will find detailed instructions on how to use the tool. If you encounter any issues or have any questions when using PACE, please email us at figures@plos.org.

Data Requirements:

Please note that, as a condition of publication, PLOS' data policy requires that you make available all data used to draw the conclusions outlined in your manuscript. Data must be deposited in an appropriate repository, included within the body of the manuscript, or uploaded as supporting information. This includes all numerical values that were used to generate graphs,

histograms etc.. For an example in PLOS Biology see here: http://www.plosbiology.org/article/info%3Adoi%2F10.1371%2Fjournal.pbio.1001908#s5.

Reproducibility:

To enhance the reproducibility of your results, we recommend that you deposit your laboratory protocols in protocols.io, where a protocol can be assigned its own identifier (DOI) such that it can be cited independently in the future. Additionally, PLOS ONE offers an option to publish peer-reviewed clinical study protocols. Read more information on sharing protocols at https://plos.org/protocols?utm_medium=editorial-email&utm_source=authorletters&utm_campaign=protocols

References:

Review your reference list to ensure that it is complete and correct. If you have cited papers that have been retracted, please include the rationale for doing so in the manuscript text, or remove these references and replace them with relevant current references. Any changes to the reference list should be mentioned in the rebuttal letter that accompanies your revised manuscript.

If you need to cite a retracted article, indicate the article's retracted status in the References list and also include a citation and full reference for the retraction notice.
